# Enhanced Near-Infrared Organic Photodetectors Leveraging Core–Shell Nanotripods

**DOI:** 10.1021/acsami.5c02476

**Published:** 2025-06-02

**Authors:** Kaiwen Zheng, Baozhong Deng, Nan Chen, Clemence Chinaud-Chaix, Mona Tréguer-Delapierre, Bruno Grandidier, Renaud Bachelot, Tao Xu, Jianhua Zhang, Furong Zhu

**Affiliations:** 1 School of Microelectronics, 34747 Shanghai University, Shanghai 200444, China; 2 CNRS, Bordeaux INP, ICMCB, 27086 University of Bordeaux, UMR 5026, Pessac F-33600, France; 3 Université de Lille, CNRS, Centrale Lille, Université Polytechnique Hauts-de-France, Junia-ISEN, UMR 8520 - IEMN, Lille 59000, France; 4 Light, nanomaterials, nanotechnologies (L2n) Laboratory, CNRS UMR 7076. University of Technology of Troyes, 12 rue Marie Curie, F-10004 Troyes Cedex, France, and CNRS-International-NTU-Thales Research Alliance (CINTRA), Nanyang Technological University. 50 Nanyang Drive, Singapore 637553, Singapore; 5 Department of Physics, Research Centre of Excellence for Organic Electronics, Institute of Advanced Materials, 26679 Hong Kong Baptist University, Kowloon Tong, NT, 999077 Kowloon,Hong KongChina

**Keywords:** NIR photodetectors, organic photodetectors, core−shell nanotripods, LSPR, enhanced NIR absorption

## Abstract

Near-infrared (NIR) photodetectors are essential for diverse applications, including medical diagnostics, optical communication, and bioimaging. Traditional photodetectors, typically made from silicon and III–V semiconductors, struggle with large-area devices on precured or flexible substrates due to complex manufacturing and high costs. Organic photodetectors (OPDs), however, offer cost-effectiveness, flexibility, and a customizable spectral response. In this study, we report our effort to enhance NIR absorption in OPDs by incorporating core–shell structured PdCu@Au@SiO_2_ nanotripods (NTs) with a *D*_3h_ configuration, designed for localized surface plasmon resonance (LSPR) beyond 1000 nm. Integrating these NTs into the OPD active layer significantly boosts NIR absorption, achieving a responsivity of 0.46 A/W and a dynamic range of 145 dB at 1050 nm. NT-based OPDs show superior sensitivity over the control OPD and a silicon photodetector at wavelengths of over 1000 nm. This improvement is due to the synergistic effects of LSPR and omnidirectional scattering from the PdCu@Au@SiO_2_ NTs, enhancing carrier generation and extraction. The improved performance highlights their potential for advanced applications such as long-range photoplethysmography and visual line-of-sight communication systems.

## Graphical abstract

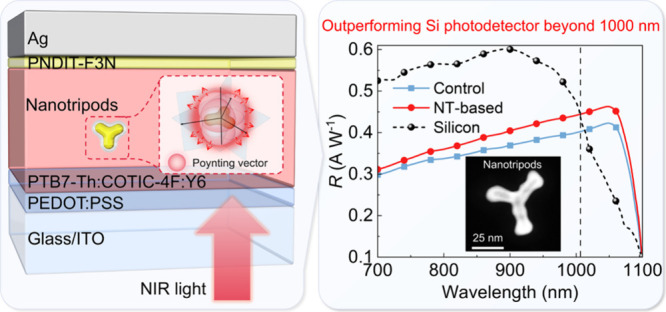

## 1 Introduction

Near-infrared (NIR) photodetectors have garnered significant attention due to their wide range of uses in medical surveillance, optical communication, artificial vision, and bioimaging.^1–5^ These applications benefit from the ability to support long-range signal transmission and enhance precision in optical systems, which are crucial for optical communication, imaging analysis, and sensing technologies. Currently, commercial photodetectors are predominantly fabricated from silicon. However, silicon-based photodetectors face challenges such as complex manufacturing processes and a limited spectral response at wavelengths over 1000 nm.^6^ Photodetectors utilizing III–V semiconductors excel in NIR detection performance but are hindered by costly manufacturing processes and limitations in flexible or conformal optical applications. The quantum dot (QD)-based photodetectors show promise for NIR detection at longer wavelengths. However, they face synthesis challenges and often include potentially hazardous elements, raising environmental and safety concerns that remain unresolved.^7–10^

Organic photodetectors (OPDs) have shown potential to serve as a promising option to overcome these limitations, leveraging their solution-processability, low cost, flexibility, and tunable spectral response.^11–15^ The advancements in OPDs are largely attributed to the fast progress in narrow band gap nonfullerene acceptors (NFAs), which have significantly extended the absorption range into the NIR spectrum.^16–19^ Recent studies have demonstrated that NIR-OPDs can achieve a specific detectivity (D*) exceeding 10^12^ Jones even beyond the 1.4 μm wavelength range.^20–22^ A common approach to enhance light absorption in OPDs involves using a thick organic photoactive layer. However, this approach has technical limitations, such as increased charge trap density, lower external quantum efficiency (EQE), and slower response speed.^23^ This trade-off between optical absorption and electrical performance presents a significant challenge in developing high-performance OPDs, particularly for applications requiring high sensitivity in the NIR range. Therefore, it is necessary to boost NIR light absorption in the OPDs without compromising their electrical characteristics.

To address this issue, various optical engineering approaches have been utilized to enhance the absorption of OPDs, e.g., using Bragg reflectors,^24^ photonic crystals,^25,26^ etc., aiming for a well-balanced photoresponse and performance.^27^ Among these methods, the use of plasmonic metal nanostructures (MNS) to improve light absorption through localized surface plasmon resonance (LSPR) is particularly effective.^28–33^ However, two critical factors must be considered when incorporating MNS into the OPDs. First, MNS embedded in the organic active layer can suffer from exciton quenching at their surface, necessitating a thin dielectric passivation layer to prevent charge recombination and improve device stability.^34,35^ Second, the alignment of the LSPR peak position with the absorption range of NFAs is essential to enhancing light absorption at specific NIR wavelengths. Most LSPR effects obtained with MNS have been limited to the visible region due to the constraint of high free carrier concentration.^36–38^ Although enlarging the geometric size of MNS can redshift the LSPR peak into the NIR region, larger MNS may disrupt the morphology of the organic active layer, making the design of MNS configuration crucial.^39^

MNS with branched structures exhibit unique optical properties due to their higher specific surface area.^40–42^ For example, multipod-planar MNS with a *D*_3h_ symmetry feature possess a horizontal symmetry plane and multiple rotational axes.^43^ The high degree of spatial symmetry allows the *D*_3h_ configuration of MNS to serve as nanoscatterers, enabling omnidirectional scattering of incident light and thereby enhancing absorption. This results in isotropic scattering enhancement, translating into longer optical paths.^44–46^ Such omnidirectional characteristics provide more efficient photon absorption enhancement while reducing the angular dependence of OPDs on the incident light’s angle,^47^ benefiting the spectral response of OPDs through a synergistic LSPR effect, especially in the NIR range.

In this work, we report our effort to achieve NIR absorption enhancement in the OPDs by incorporating core–shell *D*_3h_ structured PdCu@Au@SiO_2_ nanotripods (NTs) embedded in the photoactive layer. The NTs are designed to enable LSPR beyond 1000 nm, with a Poynting vector from NT scattering within the bulk heterojunction (BHJ) layer. We demonstrate that the use of PdCu@Au@SiO_2_ NTs facilitates efficient carrier generation and extraction in the presence of OPDs, yielding a responsivity, *R*(λ), of 0.46 A/W at 1050 nm, outperforming silicon photodetectors. Besides, a broader linear dynamic range (LDR) of 145 dB is achieved, enabling the detection of NIR light with a low intensity of 1.42 nW/cm^2^ at 1050 nm. The PdCu@Au@SiO_2_ NT-based OPDs exhibit superior sensitivity compared with control OPDs without NTs and silicon photodetectors, especially at wavelengths over 1000 nm. This is demonstrated through long-range photoplethysmography (PPG) and visual line-of-sight (VLOS) systems, highlighting their potential for applications in healthcare, automotive, and optical communication.

## 2 Results and Discussion

### 2.1 Design and Synthesis of PdCu@Au@SiO_2_ NTs

Multipod-type MNS, such as NTs, offer unique advantages over traditional configurations such as spheres and rods, as outlined in Table S1. These advantages include a highly tunable NIR response, enhanced hot-spot effects due to their branched structure, and omnidirectional scattering capabilities, making them highly effective for advanced optical applications. To enhance the NIR absorption of OPDs, this work aims at designing PdCu@Au@SiO_2_ NTs with an LSPR extinction wavelength beyond 1000 nm. Therefore, finite element method (FEM) simulations were employed to investigate the relationship between the LSPR extinction wavelength and the geometric dimensions of the NTs, providing guidance to achieve precise control over the LSPR. The contour maps of the LSPR scattering intensity plotted against the LSPR extinction wavelength and the width of the NTs with a fixed length of 30 nm, and the length of the NTs with a fixed width of 14 nm, are shown in Figure 1a,b, respectively. Based on both simulations, the LSPR extinction wavelength can be adjusted beyond 1000 nm by changing the geometrical parameters. The normalized extinction spectra as a function of the LSPR extinction wavelength are shown in Figure 1c, revealing two extinction peaks for different NTs with four different branch widths of 10, 12, 14, and 16 nm. The one in the visible wavelength range originates from the PdCu core, whereas the main peak in the NIR wavelength range is produced by the Au and SiO_2_ bishell. In this work, the main LSPR peak of the PdCu@Au@SiO_2_ NTs is required to enhance the spectral response over the NIR range, e.g., at 1050 nm. In accordance with the FEM simulation, NTs with an LSPR peak wavelength of 1053 nm were synthesized, featuring a length of 30 nm and a width of 14 nm, highlighted by the yellow line in Figure 1c.

**Figure 1:**
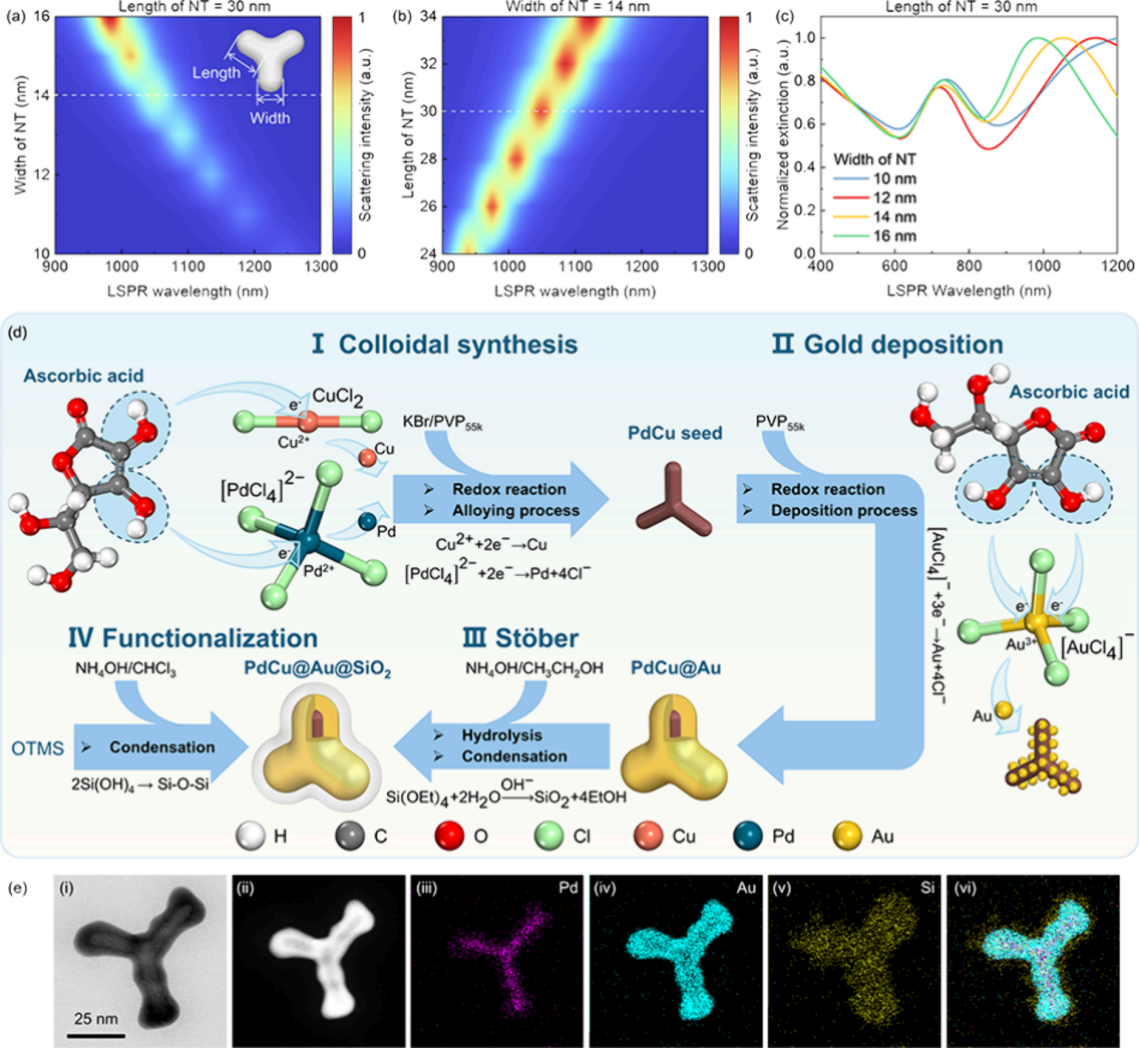
(a) Contour plot showing the relationship between NT width and LSPR wavelength. (b) Contour plot illustrating the relationship between NT length and LSPR wavelength. Inset: schematic diagram of the NT structure. (c) Normalized LSPR peak positions as a function of wavelength for NTs with a length of 30 nm and widths of 10, 12, 14, and 16 nm. (d) Synthesis scheme of PdCu@Au@SiO_2_ NTs used in this study. (e-i) TEM image of the NT. (e-ii) HAADF image of the NT. (e-iii) EDS mapping of Pd. (e-iv) EDS mapping of Au. (e-v) EDS mapping of Si. (e-vi) Superimposed EDS mapping showing the distribution of Pd, Au, and Si in the NT.

The synthesis of the PdCu@Au@SiO_2_ NTs was achieved through a multiple-step process consisting of colloidal synthesis, gold deposition, and the Stöber process, as illustrated in Figure 1d. Initially, the synthesis of the PdCu NTs begins with the reduction of Pd^2+^ and Cu^2+^ by ascorbic acid, resulting in the formation of small, plate-like seeds measuring a few nanometers. Each seed contains a single planar defect, which serves as a nucleation site for the subsequent growth. In detail, Br^–^ selectively binds onto the ⟨100⟩ facets of the seeds, while PVP_55K_ interacts with the metallic precursors, binding to the PdCu seed surface by coordinating through the nitrogen and oxygen lone pairs in the pyrrolidone ring. Such binding preferences for specific facets are thought to hinder the growth of facets and promote elongation along the ⟨211⟩ direction, resulting in the distinctive tripod structure. Next, Au^0^ atoms uniformly coat the surface of the PdCu seed due to the high reactivity of the seed surface, forming a core–shell structure PdCu@Au NT. Subsequently, tetraethyl orthosilicate was introduced into the PdCu@Au NTs colloidal dispersion, forming a thin silica coating on the exterior of the PdCu@Au NTs. Finally, to address the relatively rough surface of the silica due to the presence of polymer chains on the gold surface, an amphiphilic molecular ligand, octadecyltrimethoxisilane, was introduced to functionalize the PdCu@Au@SiO_2_ NTs, improving the stability of the NTs in chloroform, which can be confirmed by the identical extinction spectra measured for the NTs after 8 months of storage, as shown in Figure S1. The transmission electron microscopy (TEM) images measured for the NTs also verify the successful synthesis and excellent dispersion of the NTs in chloroform, as shown in Figure S2a,b.

The size distribution of the NTs, with a branch length of 30 ± 6 nm and a branch width of 14 ± 2 nm, is shown in Figure S3a,b, agreeing well with the FEM simulation results, demonstrating the achieved precision in controlling particle morphology. The relatively narrow size distribution ensures that the average LSPR peak position of the NTs remains closed at the designed wavelength of 1050 nm, which is crucial for achieving enhanced NIR absorption. This is further supported by the precisely controlled LSPR peak position of the NTs. TEM images measured for the NTs with three equiangular branches and the PdCu seed reveal a complete coverage of the Au inner shell and SiO_2_ outer shell, as shown in Figure 1e-i and Figure 1e-ii, confirming the successful synthesis of the core-bishell structure of PdCu@Au@SiO_2_ NTs. Energy-dispersive spectroscopy (EDS) mappings of the NTs confirm the presence of a uniform 2 nm-thick silica outer shell, as illustrated in Figures 1e-iii to 1e-vi. This optimal shell is designed to prevent undesirable quenching in the organic active layer.^48,49^

### 2.2 NT-Based OPDs with Enhanced NIR Photoresponse

The PdCu@Au@SiO_2_ NTs with customized LSPR were incorporated in the organic active layer, aiming to improve the light harvesting and optimize the light scattering in the NIR region. The schematic cross-sectional view of the OPDs comprising a layer configuration of glass/ITO/PEDOT:PSS/active layer/PNDIT-F3N/Ag is shown in Figure 2a. A 40 nm-thick PEDOT:PSS hole-transporting layer and a 10 nm-thick PNDIT-F3N electron-transporting layer are used to assist in charge collection in the OPDs. A 120 nm thick Ag was employed as the cathode. The organic active layer is composed of a donor PTB7-Th and two acceptors COTIC-4F and Y6 with a thickness of around 200 nm. The molecular structures of the functional materials used in this work are shown in Figure S4. The schematic energy-level diagram of the functional materials is shown in Figure 2b, presenting a cascade alignment of the highest occupied molecular orbital levels that facilitates efficient hole transfer in the OPDs. As shown in Figure S5, the donor and acceptors exhibit complementary absorption over the wavelength range of 300 to 1100 nm. Notably, Y6 features a larger optical band gap than COTIC-4F, which helps minimize thermal excitation of charge carriers.^50^ As shown in Figure S6, by integrating the NTs in the active layer with a concentration of 0.15 wt %, a 5% increase in absorption is observed at the wavelengths beyond 1000 nm.

**Figure 2:**
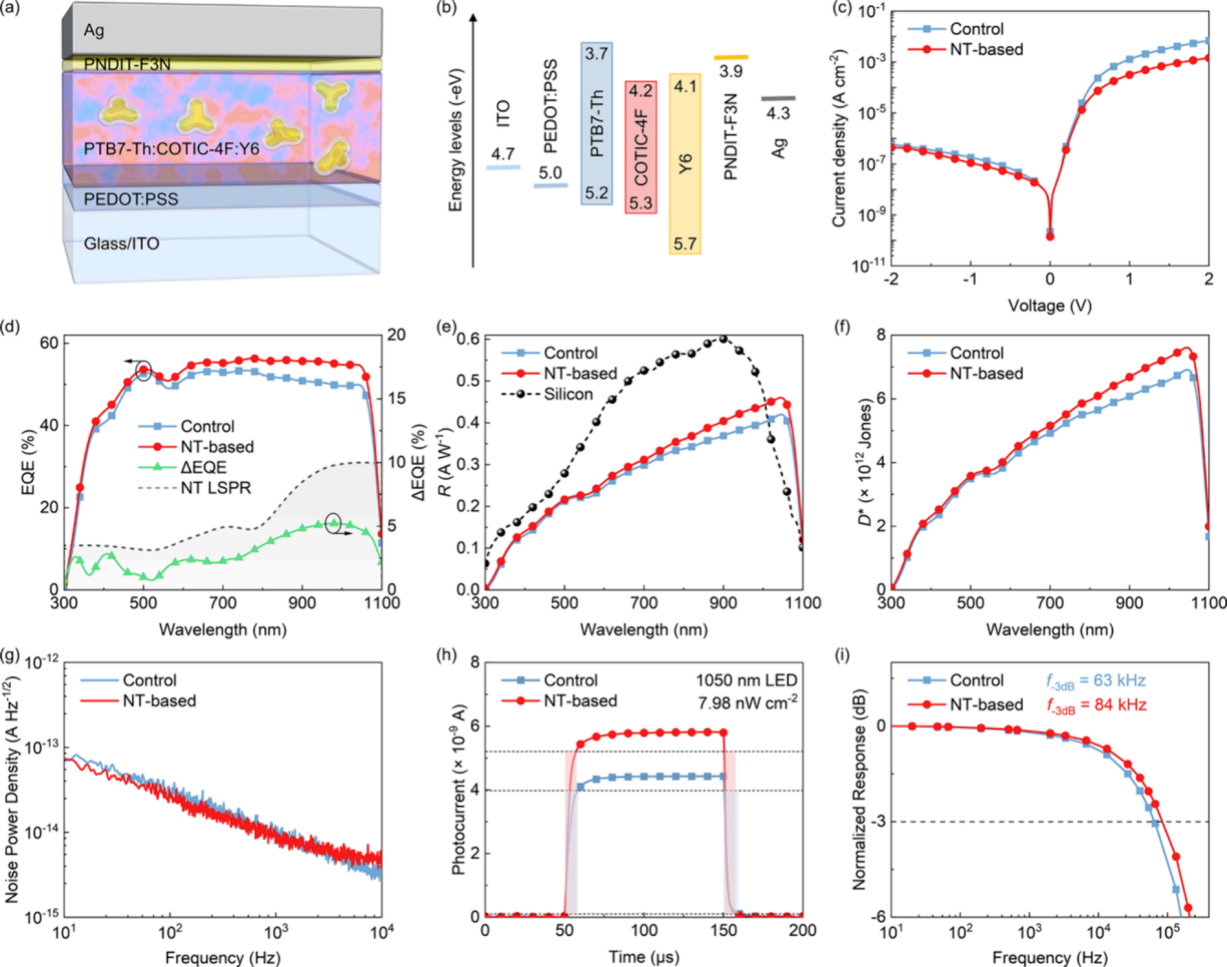
(a) Schematic cross-sectional view of an NT-based OPD. (b) Schematic representation of the energy levels of the functional materials used in the OPDs. (c) *J*_d_–*V* characteristics of the OPDs. (d) EQE spectra and the difference in EQE between NT-based and control OPDs, measured at −0.1 V. The LSPR extinction spectrum of the NTs (Gray dashed line) is also shown for comparison. (e) *R*(λ) spectra derived from EQE, measured at −0.1 V, with the corresponding *R*(λ) for a Hamamatsu silicon photodiode (black line) under the same conditions for comparison. (f) D* spectra derived from EQE and *J*_d_ measured at −0.1 V. (g) Noise spectral density measured for NT-based and control OPDs at −0.1 V. (h) Time response of the OPDs without bias using a 1050 nm LED light source. (i) *f*_–3 dB_ measured for NT-based and control OPDs without bias using a 1050 nm LED light source.

To verify the optical enhancement, a series of NT-based OPDs were fabricated with different NT concentrations incorporated into the active layer. It shows that an evident enhancement in the NIR response is realized in the NT-based OPDs, with an optimal concentration of NTs (0.15 wt %) in the BHJ layer, as shown in Figure S7. The results are summarized in Table S2. The dark current density–voltage (*J*_d_-*V*) characteristics and EQE spectra measured for the NT-based OPDs (0.15 wt %) and the control OPD without NTs are shown in Figure 2c,d. *J*_d_ measured for the NT-based and control OPD under reverse bias is almost the same. However, an increase in *J*_d_ was observed for the NT-based OPD with a higher NT concentration of 0.20 wt %, leading to a decrease in EQE as compared to that of the OPD with an optimal NT concentration of 0.15 wt %. The increase in *J*_d_ in the NT-based OPD with a high NT concentration is due to the aggregation of NTs in the active layer, which is not favorable for retaining a smooth film morphology and charge transport, although the EQE of the NT-based OPDs with a high NT concentration of 0.20 wt % is still higher than that of the control OPD, caused by the NT-induced enhanced NIR absorption and LSPR effects.^51^ In the OPD with an optimal NT concentration of 0.15 wt %, NTs can be considered as an optical amplifier, assisting in NIR absorption through scattering and LSPR effects with negligible adverse effects on charge transport. The atomic force microscopy (AFM) measurements show that an optimal NT-based organic blend layer has a root-mean-square (RMS) roughness of 1.31 nm, which is comparable to that of a control organic blend layer (1.21 nm), as shown in Figure S8. A good dispersion of NTs in the organic active layer is clearly seen in the TEM image measured for the organic blend layer with an optimal NT concentration (0.15 wt %), as shown in Figure S9.

At −0.1 V, a *J*_d_ of 1.14 × 10^–8^ A/cm^2^ was obtained for the NT-based OPD, which is almost the same as that of a control OPD without NTs (1.15 × 10^–8^ A/cm^2^), suggesting that incorporating an optimal amount of the NTs in the active layer, e.g., 0.15 wt % in this work, does not cause any undesired leakage current. A 54.16% EQE was obtained for an NT-based OPD at 1050 nm, operated at −0.1 V, which is ∼10% higher than that of a control OPD operated under the same conditions (49.47%). The increased spectral response of the NT-based OPD at 1050 nm is mainly due to the improved NIR absorption enhancement, induced by the omnidirectional scattering and LSPR effects enabled by the dispersed NTs in the BHJ active layer. The impact of the 2 nm-thick SiO_2_ outer shell on reducing exciton quenching at the metal–organic semiconductor interface was further examined by evaluating the performance of OPDs prepared with a BHJ incorporating NTs at the same concentration but without the SiO_2_ shell. The results, depicted in Figure S10, clearly demonstrate that OPDs with NTs lacking the SiO_2_ shell exhibited a reduced EQE and an increased *J*_d_ compared to the control OPD. These findings provide compelling experimental evidence of the SiO_2_ outer shell’s effectiveness in preventing exciton quenching in the active layer. *R*(λ) can be determined using the expression^52^

$$R=\frac{\mathrm{EQE}\times \lambda }{1240}$$ 1

where λ is the wavelength. An *R* of 0.46 A/W is obtained for the NT-based OPD at 1050 nm, which is higher than that of a control OPD (0.42 A/W) and a typical silicon photodetector (0.25 A/W), as shown in Figure 2e.^48^ The D* of the photodetector is wavelength dependent, which is related to *R*(λ) and noise spectral density *S*_*n*_:^53,54^

$$D*=\frac{\sqrt{A}\times R(\lambda )}{S_{n}}$$ 2

where *A* is the active area of the OPDs, 0.05 cm^2^ in this work. A D* of >7.5 × 10^12^ Jones at 1050 nm was obtained for an NT-based OPD operated at −0.1 V, as shown in Figure 2f, which is higher than that of the control OPD (6.90 × 10^12^ Jones) at 1050 nm. The *S*_n_ of the control and the NT-based OPD in the dark, operated under −0.1 V, was also analyzed to gain an insight into the improved understanding of the enhanced performance of the NT-based OPDs. It shows that the *S*_n_ of the OPDs decreases with the frequency over the frequency range from 0.1 to 10^4^ Hz, as shown in Figure 2g, indicating the frequency-dependent behavior. An *S*_n_ of 2.37 × 10^–14^ A/Hz^1/2^ is obtained for the NT-based OPD at 100 Hz, which is similar to that of the control OPD operated under the same condition (2.69 × 10^–14^ A/Hz^1/2^). The transient photoresponse characteristics measured for the NT-based and control OPDs operated at −0.1 V, under illumination of an NIR (1050 nm) LED light source with an irradiation of 7.98 nW/cm^2^ are shown in Figure 2h. The rise time (τ_r_) and fall time (τ_f_) of OPDs are characterized by the duration required for the transient photocurrent to ramp up from 10 to 90% of its peak value and to decay from 90 to 10% of its lowest value, respectively.^56^ The τ_r_ and the τ_f_ are slightly reduced for the NT-based OPD. Due to the random distribution of NTs within the active layer, some are positioned near the charge-transporting layer, which facilitates the rapid transfer of the carriers generated around these NTs into the charge-transporting layers. The LSPR effect of NTs results in a faster carrier collection and diffusion within the active layer, thereby improving the response speed.^57,58^ Next, the −3 dB cutoff frequency (*f*_–3 dB_) was also investigated to determine the usable bandwidth, where the output amplitude drops by 3 dB. As shown in Figure 2i, consistent with the τ_r_ and the τ_f_, the NT-based OPD possesses a higher *f*_–3 dB_ of 84 kHz than that of the control OPD, confirming the effectiveness of integration of the NTs. In addition, the stability test was performed for the NT-based OPD, which was encapsulated and stored in air. We also conducted an aging test to analyze the photoresponse behavior of the encapsulated NT-based OPDs in air. The results are shown in Figure S11. The NT-based OPD retains 94.4% of its initial photocurrent after a 15-day aging test, indicating that the core–shell PdCu@Au@SiO_2_ NTs are well-suited for enhancing NIR absorption and ensuring the operational stability of NIR-OPDs. Additionally, the fabrication process demonstrates excellent reproducibility, with a batch-to-batch variation of ± 1.0 × 10^–10^ A/cm^2^ in *J*_d_ and ± 0.39% in EQE at 1050 nm for the OPDs operated at −0.1 V. These results were consistent across different batches using identical fabrication processes and experimental conditions. A summary of the device performance obtained for the NT-based and control OPDs is given in Table 1, demonstrating the advantage of incorporating core–shell PdCu@Au@SiO_2_ NTs for enhanced NIR spectral response in OPDs.

**Table 1:** Performance of the Control and the NT-based OPDs^a^

device	EQE [%]	*R* [A/W]	*J*_d_ [A/cm^2^]	D* [Jones]	τ_r_ [μs]	τ_f_ [μs]	*f*_–3 dB_ [kHz]
control	49.47 ± 0.32	0.42	(1.15 ± 0.01) × 10^–8^	6.90 × 10^12^	8.04	8.57	63
NT-based	54.17 ± 0.39	0.46	(1.14 ± 0.01) × 10^–8^	7.58 × 10^12^	6.91	7.04	84

a The results were obtained for the OPDs operated at −0.1 V, under illumination of NIR (1050 nm) light, and averaged from the measurement of 10 OPDs.

### 2.3 Effect of PdCu@Au@SiO_2_ NTs on OPD Performance

To gain physical insight into the enhanced performance of the OPDs, the LSPR effect and the scattering behavior of the PdCu@Au@SiO_2_ NTs have been systematically investigated. It shows that the enhancement in EQE (∼10%) exceeds that of absorption (∼5%), which can be ascribed to the omnidirectional scattering effect of NTs. As shown in Figure 3a, the NT constitutes a type of MNS featuring a *D*_3h_ configuration, which encompasses a horizontal symmetry plane and a *C*_3_ axis perpendicular to the symmetry plane, along with three *C*_2_ rotational axes orthogonal to the *C*_3_ axis. As depicted by the red sphere in Figure 3a, the enhanced Poynting vector in all orientations implies that NTs act like omnidirectional nanoscatterers, which have the capacity of scattering incident photons in all directions, resulting in an isotropic scattering enhancement. It extends the optical path within the active layer and, thereby, improves NIR photon absorption. The omnidirectional scattering effect was quantified and analyzed using the equation below:

Π⃗(r⃗,ω)=12Re{E⃗(r⃗,ω)×H*⃗(r⃗,ω)} 3

where *H⃗* is the magnetic field. Π⃗ is the Poynting vector, which was computed numerically along the white-dashed ellipsoid, with the red line indicating the positions of the Poynting vector emanating from points of the ellipsoid. The wavelength-dependent refractive index and the extinction coefficient of all of the materials used in the OPDs are shown in Figure S12a,b. The electric field distribution in the OPDs with NTs embedded in the active layer at various rotation angles under excitation at 1050 nm (longitudinal polarization), along with the corresponding Poynting vectors, is shown in Figure 3b. First, the near-field enhancement around the NTs can be observed. Second, the Poynting vector is generated, facilitating enhanced NIR absorption in the OPDs. It shows that the Poynting vector persistently demonstrates enhancement at resonant wavelengths in all directions. Commonly, incident light perpendicularly enters the OPDs and passes through the active layer, resulting in limited photon absorption along the vertical direction. Owing to the omnidirectional scattering effect introduced by the NTs, photons are scattered in directions nearly parallel to the horizontal plane, effectively prolonging the light path within the active layer. Statistically, the NTs produce a far-field brightness that appears uniform from all viewing angles. Consequently, such omnidirectional light propagation is expected to increase, significantly boosting the EQE. More importantly, the simulation shows that the Poynting vector is extended to the charge-transporting layers, indicating that the omnidirectional scattering effect of NT facilitates the generation and dissociation of carriers in the entire active layer, which will enhance the carrier collection and extraction efficiency.

**Figure 3:**
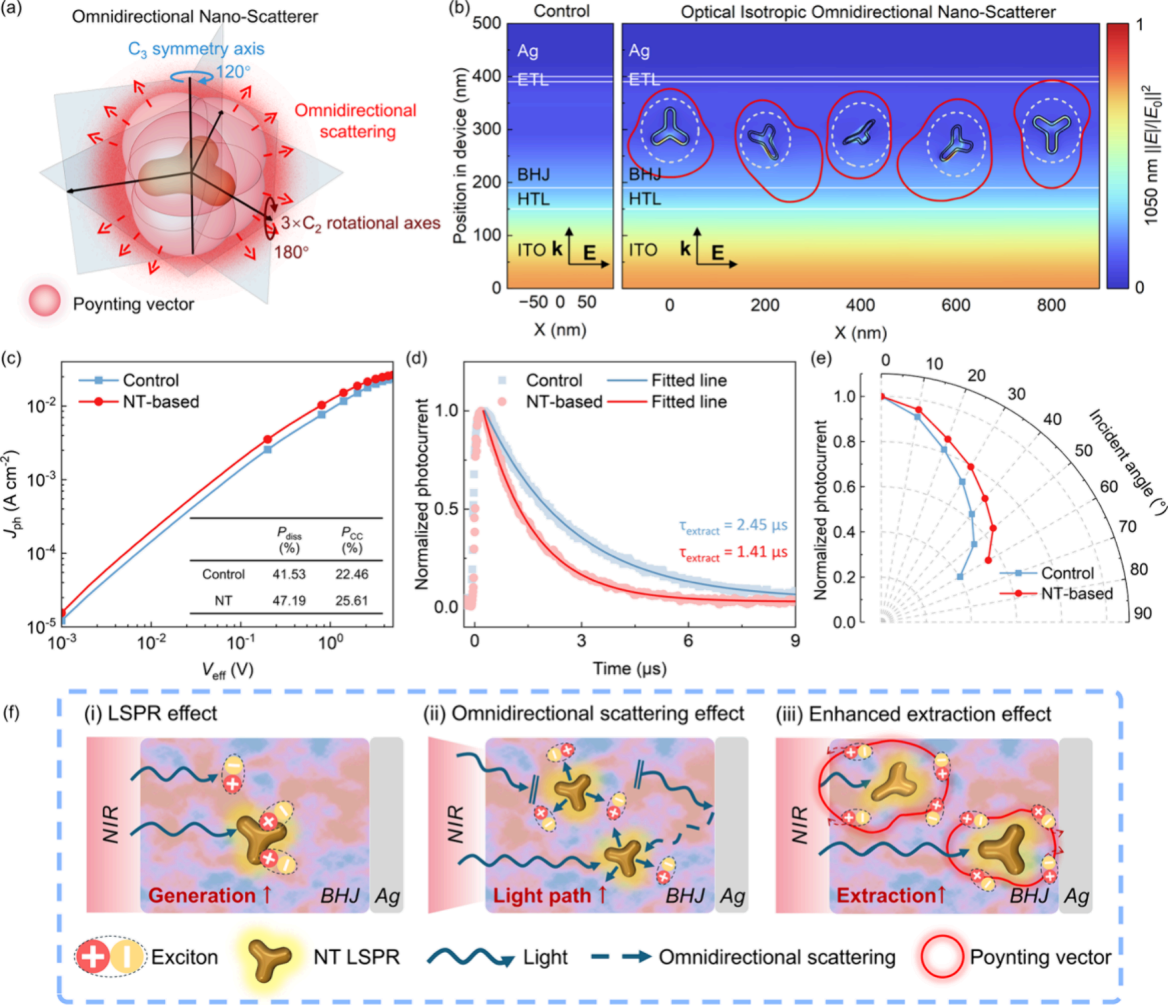
(a) Schematic diagram illustrating the scattering of an NT with a *D*_3h_ configuration. (b) Electric field intensity distributions in OPDs without (left) and with (right) NTs in the active layer, shown at various rotation angles under excitation at 1050 nm. The red locus illustrates the scattering with the amplitude of the Poynting vector Π⃗ along the white-dashed integral surfaces of the NTs. (c) *J*_ph_–*V*_eff_ characteristics. (d) TPC characteristics. (e) Normalized photocurrent as a function of the angle of incident light (1050 nm) for NT-based and control OPDs. (f-i) Schematic diagram depicting the LSPR effect. (f-ii) Schematic diagram depicting the omnidirectional scattering effect. (f-iii) Schematic diagram depicting the enhanced extraction effect realized by NTs for enhanced NIR light absorption in OPDs.

To validate the NT-induced effect, a series of characteristics were performed. The photocurrent density-effective voltage (*J*_ph_–*V*_eff_) characteristics measured for the NT-based and control OPDs are shown in Figure 3c. *J*_ph_ = *J*_1_ – *J*_d_, where *J*_1_ is measured under an AM of 1.5G. *V*_eff_ is obtained as *V*_eff_ = *V*_1_ – *V*_app_, where *V*_1_ is the voltage where *J*_ph_ is 0 and *V*_app_ is the applied bias. As the *J*_ph_ of both devices saturates nearly at the same *V*_eff_, an improved exciton dissociation probability (*P*_diss_) as well as the exciton collection probability (*P*_CC_) can be observed for the NT-based OPD. The *P*_diss_ increases from 41.53 to 47.19%, while the *P*_CC_ increases from 22.46 to 25.61%. Next, the transient photocurrent (TPC) characteristics were carried out for both devices. The NT-based OPD exhibits a shorter charge extraction time (1.41 μs) compared to the control OPD (2.45 μs), indicating an improved charge extraction efficiency and a reduced trap-assisted carrier recombination, which can be attributed to the enhanced built-in potential and the carrier collection (Figure 3d).^59^ Such a result is consistent with the variation of *V*_OC_ with the light intensity (*P*) (Figure S13). Based on the relationship *V*_OC_ ∝ *n*(*kT*/*q*) ln *P*, the NT-based OPD exhibits a slope of 1.184 kT/q, which is notably smaller than the 1.349 kT/q measured for the control OPD, leading to a reduction in defect-assisted recombination. Meanwhile, the exciton recombination behavior of NT-based OPDs approximates that of silicon photodiodes more closely, which is conducive to potential applications. In addition, a set of photocurrent measurements under a 1050 nm LED at different incident angles was performed. The normalized photocurrent as a function of incident angle is plotted in Figure 3e. When the angle of incident light increases to 60°, NT-based OPDs have the capacity to maintain approximately 55% of the original photocurrent at normal incidence, which is higher than that of the control OPD (40%). Thus, the increased photocurrent demonstrates that NTs can enhance the optical path, leading to improved NIR light absorption across different incident conditions.

The schematic diagrams illustrating the NT-assisted NIR absorption enhancement in the active layer are shown in Figure 3f. The enhanced NIR absorption in the NT-based OPDs is associated with the effective photon absorption processes via the NT-induced LSPR and the omnidirectional scattering effects in the organic active layer. The theoretical simulation reveals that when NTs are positioned relatively at the center of active layer, the Poynting vector still can be extended toward the charge-transporting layer, realized by the strong scattering effect, suggesting that the carrier generation and dissociation at the donor–acceptor interface near the charge-transporting layer can also be enhanced. The reduced carrier transporting distance, combined with enhanced built-in potential, facilitates more efficient extraction and collection of charge carriers, ultimately leading to improved OPD performance.

A series of carrier dynamics analyses were performed to systematically elucidate the mechanisms underlying the performance enhancements induced by the incorporation of NT, and the results are summarized in Tables S3 and S4. The hole mobility (μ_h_) and electron mobility (μ_e_) were obtained using the space-charge-limited current (SCLC) measurement and calculated based on the Mott–Gurney law:

$$\begin{array}{c} J=\frac{9}{8}\varepsilon_{0}\varepsilon_{r}\mu \frac{V^{2}}{d^{3}} \end{array}$$ 4

where ε_0_, ε_r_, μ, and *d* represent the vacuum permittivity, the relative dielectric constant, the mobility, and the thickness of the active layer (200 nm), respectively. The NT-based OPD shows a slightly higher μ_h_ of 4.30 × 10^–5^ cm^2^/V/s and μ_e_ of 2.72 × 10^–5^ cm^2^/V/s than that of the control OPD (μ_h_ of 3.85 × 10^–5^ cm^2^/V/s and μ_e_ of 2.28 × 10^–5^ cm^2^/V/s). A more favorable ratio of μhμe 1.58 is achieved for the NT-based OPD as compared to that of the control OPD (1.69), as shown in Figure 4a. This suggests enhanced carrier mobility and improved pathways for carrier transport, resulting in a smaller space-charge region.^60^ Hole trap density (*N*_t_) of the OPDs was examined using the following relationship:

$$\begin{array}{c} V_{TFL}=\frac{qN_{t}d^{2}}{2\varepsilon_{0}\varepsilon_{r}} \end{array}$$ 5

where *V*_TFL_ is the trap-filled limit voltage. The SCLC results and the corresponding fitted lines of the hole-only devices made with and without NTs are shown in Figure S14. It is evident that NT-based OPD possesses a smaller *V*_TFL_, thereby resulting in a comparatively suppressed *N*_t_ of 2.29 × 10^15^ cm^–3^, indicating a reduction of nonradiative recombination loss and a lower leakage current, which can be attributed to the hot-carrier effect.^61^ The electrochemical impedance spectroscopy (EIS) was also performed for both devices. As shown in Figure 4b, a lower fitted transfer resistance derived from the Nyquist plots is observed for the NT-based OPD, indicating a reduced charge injection barrier, which is consistent with the better carrier mobility.^62^ The photo fluorescence (PL) spectra measured for the two ITO/HTL/organic active layer samples are shown in Figure S15. The pronounced quenching is observed in the NT-based film, suggesting an improved hole extraction. The charge carrier dynamics was further examined through time-resolved photoluminescence (TRPL) characteristics, as illustrated in Figure 4c. The lifetime was calculated by the double exponential fitting method. The NT-based OPD possesses a shorter exciton lifetime (τ_1_ = 0.02 ns, τ_2_ = 1.52 ns) than that of the control OPD (τ_1_ = 0.11 ns, τ_2_ = 4.89 ns), indicating that the addition of NTs can enhance exciton extraction while reducing radiative recombination between HTL and the active layer.^63^

**Figure 4:**
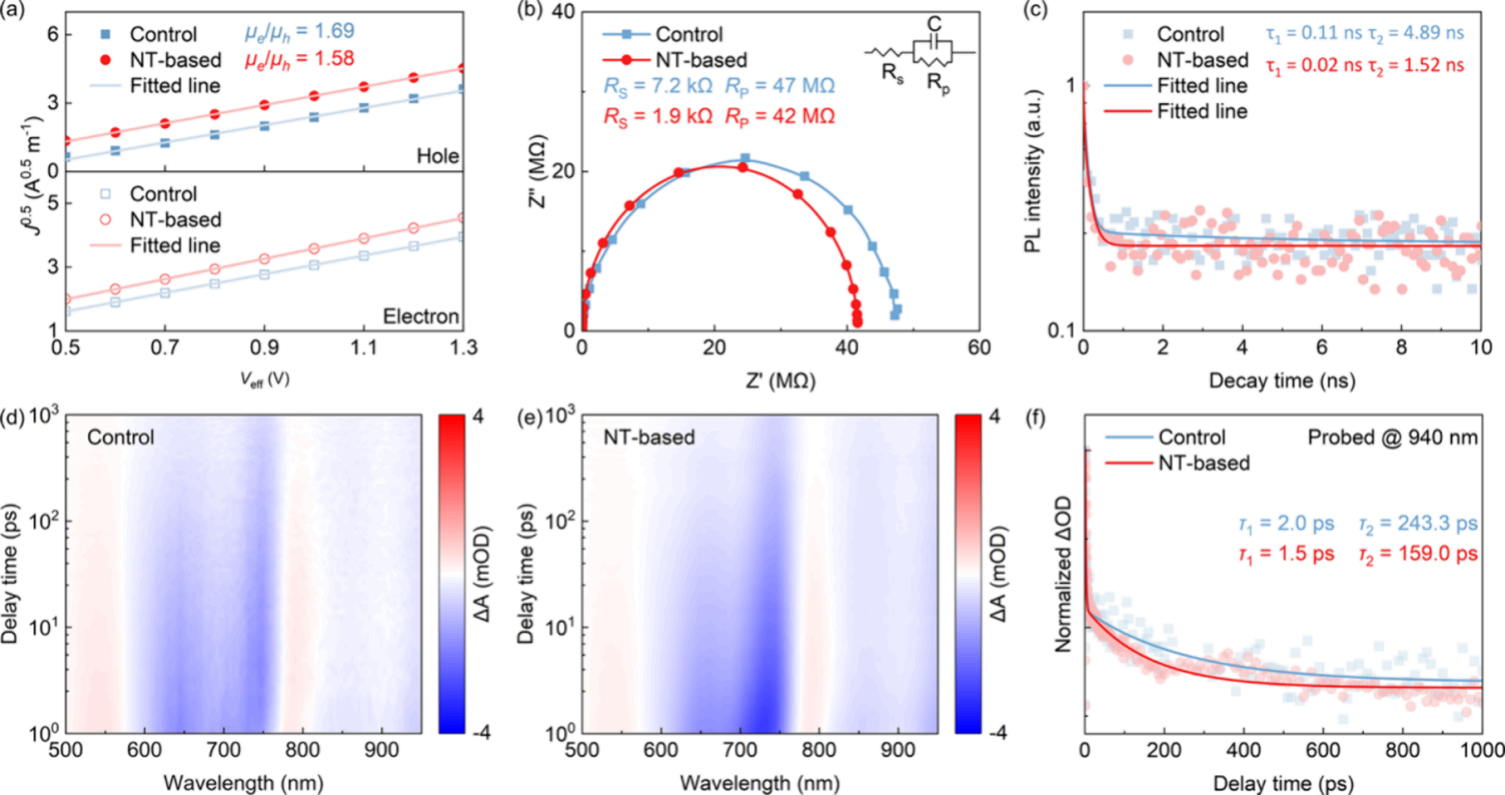
(a) SCLC measurements. (b) Nyquist plot in the dark measured at open-circuit condition. (c) TRPL spectra measured for NT-based and control OPDs. (d) 2D TA contour map for BHJ films without NTs. (e) 2D TA contour map for BHJ films with NTs. (f) GSB signals probed at 940 nm for BHJ films without and with NTs.

The capacitance–voltage (1/*C* ^2^–*V*) characteristics and the corresponding Mott–Schottky analysis were then performed to examine the EQE enhancement by the addition of NTs.^64^ The slope of the Mott–Schottky plot derived from 1/*C* ^2^–*V* reflects the trap density (*N*_A_), which can be determined by the expression below:

$$N_{A}=\frac{-2}{q\varepsilon_{0}\varepsilon_{r}A^{2}}\left(\frac{dV}{dC^{-2}}\right)$$ 6

The depletion width (*W*)^65^ can be obtained using the following expression:

$$W=\sqrt{\frac{2\varepsilon_{0}\varepsilon_{r}\left(V_{\mathrm{bi}}-V\right)}{qN_{A}}}$$ 7

where *V*_bi_ is the built-in voltage, which can be derived from the intersection point of the fitted line of the Mott–Schottky plot and the voltage axis. The NT-based OPD exhibits a higher *V*_bi_ of 0.295 V as compared to that of a control OPD (0.274 V), as illustrated in Figure S16, indicating that photogenerated carriers are more effectively separated and collected by the electrodes on both sides, thereby contributing to the enhanced EQE. The NT-based OPD also exhibits a smaller hole trap density and a larger depletion width, revealing that the incorporation of NTs may potentially enhance the electrical performance of the OPDs, attributed to the hot-carrier effect. The transient absorption (TA) measurements were conducted to further investigate the photoinduced charge transfer dynamics. As illustrated in Figures 4d and 4e, the ground state bleaching (GSB) signal for the acceptors is beyond 940 nm (limited by the spectrometer). Compared to the control BHJ film without NTs, a more obvious signal intensity is observed for the NT-based BHJ film, indicating improved carrier transport properties. As shown in Figure 4f, the fast lifetime τ_1_ and the slow lifetime τ_2_ are calculated by fitting the GSB signals. The τ_1_ and τ_2_ obtained for the NT-based BHJ film are 1.5 and 159.0 ps, which are shorter than the ones observed for the BHJ film without NTs (2.0 and 243.3 ps). These results demonstrate a faster exciton dissociation as well as a more effective exciton extraction and collection,^66^ leading to better electrical properties and performance. In addition to the NIR absorption enhancement, the incorporation of core–shell PdCu@Au@SiO_2_ NTs also assists in charge transport through multiple pathways, leading to a simultaneous improvement in both optical and electrical properties.^67^

In addition, to verify the versatility of the dual effect induced by NTs, we fabricated the PM6:BTP-eC9-based OPDs that have been reported. The device structure of the binary OPD, the molecular structures of the organic material used in the active layer, and the energy levels of all the functional materials are depicted in Figure S17. The thickness of the active layer was also fixed at around 200 nm. Figure S18a shows that PM6 and BTP-eC9 have complementary absorption in 300–900 nm. Based on FEM simulation, NTs with a length of 26 nm and a width of 20 nm were fabricated, which led to an LSPR peak wavelength of 811 nm, and introduced into the organic active layer. A 4% increase in the absorption over the wavelength range from 800 to 850 nm is observed, as shown in Figure S18b. The characterization results are summarized in Figure S19 and Table S5, showing that an evident enhanced NIR spectral response is clearly realized in the NT-based OPD with the addition of NTs.

### 2.4 High-NIR Sensitivity of NT-Based OPDs

As the addition of NTs significantly increases the EQE while nearly unchanging *J*_d_, the weak light detection performance of the OPDs is characterized systematically. The LDR is a critical parameter that determines the spectrum of light intensities at which OPDs display a linear response. The LDR is calculated by the photocurrent measured at various light intensities based on the following expression:

$$\mathrm{LDR}=20\log\;\frac{J_{\max}}{J_{\min}}$$ 8

where *J*_max_ and *J*_min_ are the photocurrent densities recorded at the maximum and minimum light intensities, just before the response departs from linearity, respectively. As shown in Figure 5a, the NT-based OPD can maintain good linearity at a weak light intensity of 1050 nm LED light, thereby enhancing the LDR from 130 to 145 dB, which is also significantly higher than that of silicon photodiodes (96 dB). Figure 5b,c exhibits the *J*_min_ for the control OPD and the NT-based OPD, with a light intensity of 8.33 and 1.42 nW/cm^2^, respectively. At the lower light intensity, the NT-based OPD works better than the control OPD. An imaging measurement setup with a 130 × 40 mm-sized ″SHU″ mask, having a letter line width of 4.7 mm, was used to test the NT-based and control OPD under illumination of an NIR LED light source with different light intensities, as shown in Figure S20a. In this imaging setup, an LED light source is aligned with an NT-based OPD. The ″SHU″ metal mask is positioned between the light source and the OPD sensor. The photocurrent is measured by moving the mask linearly along the *X* and *Y* directions, using a computer-controlled x-y stepper motor stage. This process generates the ″SHU″ images, which vary according to the light intensity. For example, when illuminated by an NIR (1050 nm) LED light source with an intensity of 2.4 nW/cm^2^, a clear image can be produced using an NT-based OPD sensor. In contrast, a control OPD sensor fails to generate a clear image under the same conditions, as shown in Figure S20b. This highlights the significantly enhanced sensitivity of NT-based OPDs to weak light, particularly in the NIR region, making them highly suitable for NIR sensing applications.

**Figure 5:**
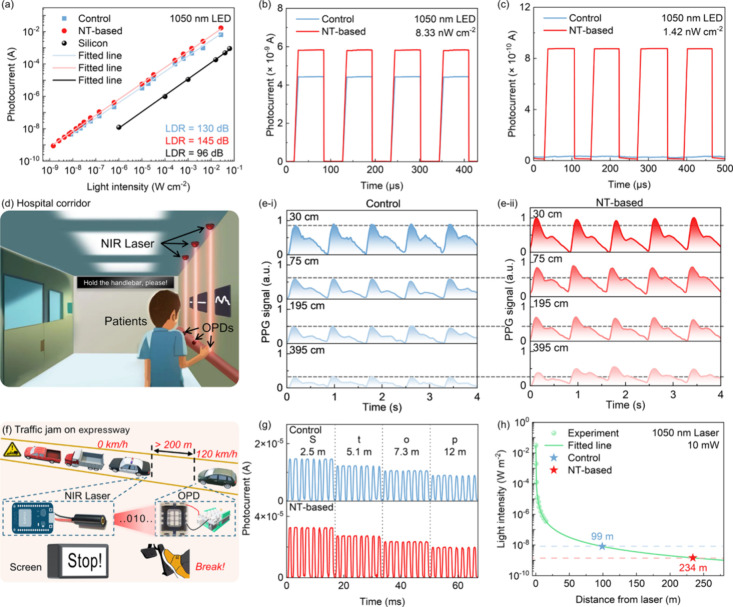
(a) LDR characteristics for NT-based and control OPDs using a 1050 nm LED light source, with LDR for a Si photodetector also shown for comparison. (b) Time response for NT-based and control OPDs without bias under illumination of a 1050 nm LED source with an intensity of 8.33 nW/cm^2^. (c) Time response for NT-based and control OPDs without bias under illumination of a 1050 nm LED source with an intensity of 1.42 nW/cm^2^. (d) Schematic diagram illustrating long-range PPG monitoring in a hospital corridor. (e-i) PPG signals measured for a control OPD using a 1050 nm laser at distances of 30, 75, 195, and 395 cm. (e-ii) PPG signals measured for an NT-based OPD using a 1050 nm laser at distances of 30, 75, 195, and 395 cm. (f) Schematic diagram illustrating a VLOS NIR communication system on a high-speed motorway. (g) Waveform of digital data for ″Stop″ received by control and NT-based OPDs at different distances using a 10 mW NIR laser (1050 nm). (h) Detectable light intensity, varied by changing the distance between the NIR laser (1050 nm) and the detector, for NT-based and control OPDs.

In addition to the superior sensitivity for low-light detection, the intensity of incident light is influenced by the distance between the light source and the detector. Therefore, NT-based OPDs have advantages in distance optical communication. To validate such a scenario, long-range PPG signal measurements of human vital signs were performed. As shown in Figure 5d, in medical environments, patients are unlikely to always carry heart rate monitoring devices. For instance, while walking in a corridor, patients can simply place their hands on the railing equipped with integrated OPDs to facilitate PPG monitoring. However, light signals carrying physiological information suffer from severe attenuation, diffraction, and scattering as they pass through human tissues, resulting in weak optical signals,^68^ while the NT-induced enhancement of NIR sensitivity helps to mitigate such limitations. Therefore, the PPG signals of one of the authors were subsequently recorded using OPDs attached to the fingertip, with a 1050 nm laser positioned at distances of 30, 75, 195, and 395 cm from the detector. As shown in Figure 5e, the collected signals were obtained without any additional amplification or filtering. It is evident that the PPG signals collected by NT-based OPDs exhibit stronger and more distinct systolic and diastolic peaks. At a detection distance of 395 cm, the control OPD produces noise peaks, making it unable to accurately reflect the patient’s heart rate. In contrast, the NT-based OPD is still capable of resolving both systolic and diastolic peaks. Such capability highlights the potential of OPDs for enabling more comfortable long-range PPG monitoring in hospital settings.

NT-based OPDs with enhanced spectral response at long wavelengths have an advantage for use in VLOS communication. Figure 5f presents the VLOS communication system based on the scenario of a high-speed motorway. Under poorly illuminated conditions, such as at night, VLOS communication enables the possibility of early warning, rendering highway traffic safer. When an accident occurs in the front vehicle, the emergency signal can be transmitted to the rear vehicle via NIR optical communication to fulfill the purpose of early deceleration. Figure 5g exhibits the waveform of digital data for “Stop” received by the control OPD and the NT-based OPD at different distances by using a 1050 nm Laser with a power of 10 mW. The signal is encoded using the American Information Interchange Standard Code (ASCII), where 1 and 0 are represented by the difference in switching current and duration. The time response curve demonstrates that the “Stop” signal can be wirelessly transmitted within 100 ms. Based on the *J*_min_ measured for the OPDs, it shows that the NT-based OPD exhibits an NIR optical communication distance of 234 m, which is significantly larger than that of the control OPD (99 m), as shown in Figure 5h, revealing the benefit of using NT-based NIR OPD for application in VLOS communication.

## 3 Conclusions

In summary, we have successfully developed NIR-OPDs with an enhanced photoresponse for wavelengths beyond 1000 nm by incorporating core–shell PdCu@Au@SiO_2_ NTs into the active layer. Guided by theoretical simulations, these NTs with a *D*_3h_ configuration and an LSPR extinction wavelength beyond 1000 nm were designed and synthesized. When integrated into the ternary active layer of PTB7-Th:COTIC-4F:Y6, the NTs significantly increased effective absorption and enhanced the NIR spectral response beyond 1000 nm. This enhancement is attributed to the combined effects of LSPR at the desired NIR wavelength range and the omnidirectional scattering properties of the NTs. The optimal NT-based OPDs, operated at −0.1 V, achieved an *R*(λ) of 0.46 A/W at 1050 nm and an LDR of 145 dB, outperforming the control OPD and a silicon photodetector. These advancements enable the detection of low NIR light intensities down to 1.42 nW/cm^2^, which is crucial for high-sensitivity photoresponse applications in health monitoring and optical communication.

## 4 Experimental Section

### 4.1 Materials

Glass/ITO substrates and SnO_2_ solution were purchased from Advanced Election Technology Co., Ltd. PEDOT:PSS solution (Clevios PVP AI 4083), CuSCN, C_60_, and BCP powder were purchased from Xi’An Polymer Light Technology Corp. MoO_3_ powder was purchased from Macklin. PTB7-Th and COTIC-4F were purchased from 1-Material Inc. PM6, BTP-eC9, Y6,, and PNDIT-F3N were purchased from Solarmer Beijing Inc. The following reagents were purchased from Sigma-Aldrich, including ascorbic acid (AA), copper­(II) chloride dihydrate (CuCl_2_·2H_2_O), potassium bromide (KBr), polyvinylpyrrolidone (PVP_55K_), sodium tetrachloropalladate­(II) (Na_2_PdCl_4_), gold­(III) chloride trihydrate (HAuCl_4_·3H_2_O), ammonium hydroxide (NH_4_OH), tetraethyl orthosilicate (TEOS), ocstadecyltrimethoxisilane (OTMS), absolute ethanol (EtOH), chloroform (CF), methanol, acetic acid, diethyl sulfide, 1,8-diiodooctane (DIO), and 1-chloronaphthalene (1-CN). All of the materials purchased were used without further purification.

### 4.2 Synthesis of the PdCu@Au@SiO_2_ NTs

Twenty mg of AA, 3 mg of CuCl_2_·2H_2_O, 300 mg of KBr, and 35 mg of PVP_55K_ were dissolved in 3 mL of ultrapure water. The mixture was heated to 80 °C in an oil bath with continuous magnetic stirring. Next, 1 mL of an aqueous Na_2_PdCl_4_ solution (19 mg/mL) was swiftly injected into the reaction medium. This solution was prepared at least 1 h in advance. The reaction proceeded at 80 °C for 2 h, after which the resulting PdCu seeds were collected via centrifugation (12000 g, 30 min) and washed three times with a solution of PVP_55K_ (2 g/L) to remove excess precursors. The PdCu seeds were then redispersed in 5 mL of an aqueous PVP_55K_ solution. Next, 700 mg of AA, 125 mg of PVP_55K_, and 250 μL of the prepared PdCu seeds were sequentially added to 25 mL of water in a 100 mL flask under vigorous stirring. After 10 min, a solution of HAuCl_4_·3H_2_O (5 mL, 0.5 mM) was introduced into the dispersion using a syringe pump at a rate of 5 mL/h. Then, the PdCu@Au colloidal suspension was concentrated by centrifugation and was redispersed in ethanol (6.2 mM (Au atoms)). Next, 11.4 μL of NH_4_OH was added to 200 μL of the suspension (Introduced in a 1.5 mL Eppendorf), followed by an ultrasound bath (25 °C) for 1 min. Then, 10 μL of TEOS solution (1%, v/v in EtOH) was added and the mixture was placed in an ultrasound bath (25 °C) and then stirred on a plate for 1 night. The silica-coated tripods dispersion was washed once with deionized water (DI water) and twice with EtOH (9000g, 10 min). The particles were then redispersed in 1 mL of ethanol, achieving a final concentration of 1.24 mM in Au atoms. Finally, 30 μL of NH_4_OH and 150 μL of OTMS solution (10%, v/v in CF) were sequentially added to 1 mL of Au@SiO_2_ dispersion (1.24 mM in Au). Finally, the tripods were stored in 1.5 mL of CF at a concentration of 0.83 mM in Au.

### 4.3 Device Fabrication

The ITO/glass substrates were cleaned sequentially with detergent, DI water, acetone, and isopropanol each for 20 min by ultrasonication. After being cleaned, the ITO/glass substrates were treated with plasma for 5 min. For the ternary system, the PEDOT:PSS solution was deposited on the ITO/glass substrate by using spin-coating at 2000 rpm for 30 s, followed by annealing at 130 °C for 10 min, to form a 40 nm-thick HTL. For the binary system, the CuSCN solution (20 mg/mL, dissolved in diethyl sulfide) was deposited on the ITO/glass substrate by spin-coating at 2000 rpm for 60 s, followed by annealing at 100 °C for 10 min. Next, the PTB7-Th:COTIC-4F:Y6 solution (30 mg/mL, 1:1.125:0.375 w/w/w, dissolved in CF with 0.9375 vol % 1-CN as additive), or the PM6:BTP-eC9 solution (16 mg/mL, 1:1.2 w/w, dissolved in CF with 0.5 vol % DIO as additive) was spin-coated on the prepared HTL at 1250 rpm (The ternary system) or 1000 rpm (The binary system) for 30 s, followed by an annealing at 100 °C for 10 min in a N_2_-filled glovebox, thus forming a ∼220 nm-thick active layer. Notice that the substrate should be kept at 60 °C before depositing the ternary solution. Afterward, for the ternary system, the PNDIT-F3N solution (1 mg/mL dissolved in methanol with 5 vol % acetic acid as cosolvent) was spin-coated on the active layer at 3000 rpm for 30 s, to form a 10 nm-thick ETL. For the binary system, a bilayer ETL consisting of C_60_ (10 nm)/BCP (8 nm) was deposited by thermal evaporation under high vacuum (5.0 × 10^–4^ Pa) with an evaporation rate of 0.5 Å/s. Finally, 120 nm-thick Ag as the upper metal electrode was deposited by thermal evaporation with an evaporation rate of 1.5 Å/s. The OPDs possess an active area of 5 mm^2^, defined by the crossover area between ITO and the upper Ag electrode.

### 4.4 Characterization

The OPDs were encapsulated, and all of the measurements were performed in air. The EQE spectra of the OPDs were measured by using a 7-SCSpec solar cell measurement system (7-STAR Co.) equipped with a calibrated Si detector. The *J*–*V* characteristic of the OPDs was measured using a 2635B Keithley source meter (Tektronix Inc.). The PPG signal and noise current were measured using an FS-Pro semiconductor parameter test system (Primarius Technology). The transient photocurrent response of the OPDs was measured using an MDO3104 oscilloscope (Tektronix Inc.). For the photocurrent performance, the OPDs were measured under a 1050 nm power-adjustable LED (Beyond Photonics Co., Ltd.) or a 1050 nm, 10 mW laser (SenTaiDa Laser Technology Co., Ltd.). The emission power of the LED and the laser at different light intensities was measured by a NOVA II spectrometer (Ophir Optronics Solutions Co., Ltd.). To generate a square-wave pulsed light for transient response, the LEDs were powered by an AFG-1022 function generator (Tektronix Inc.). The absorption spectra of the BHJ films were measured by using a UV-1800 PC spectrophotometer (MAPADA Instruments, Inc.). The optical constants and the thickness of the functional layers of the OPDs were measured by using an RC2-XI spectroscopic ellipsometer (J.A. Woollam). The PL and TRPL were measured with a Nano Finder 30A fluorescence spectrometer (Tokyo Instruments, Inc.). The impedance spectroscopy and the capacitance–voltage spectroscopy were measured by using a CS2350H potentiostat (Corrtest) with a bias of 0.1 V under dark conditions. The frequency setting for the impedance spectroscopy ranges from 10^–3^ to 10000 Hz. The voltage setting for the capacitance–voltage spectroscopy ranges from −0.2 to 1.0 V with a frequency of 100 Hz. The surface morphologies of different films were analyzed by using an SPA-400SPM AFM (Nanonavi). The TEM and EDS images were obtained by using a Themis ETEM G3 microscope (Thermofisher), and the samples were prepared on ultrathin carbon films. TA spectroscopy was performed using a home-built transient absorption spectrometer. A 1030 nm femtosecond laser (BFL-1030–20BS, BWT) is split by a ratio of 80:20, where the strong fraction is used as the pump source and the weak fraction as the probe source. The pump beam is frequency-tripled to generate a 343 nm beam, which is passed through a chopper with a frequency of 500 Hz and then focused onto the material through a convex lens. The probe beam is focused and passed through a 5 mm YAG crystal to generate a supercontinuum white light. The probe signal is collected by a fast fiber optic spectrometer synchronized with the chopper. The temporal resolution of the transient absorption spectrometer is around 350 fs, as determined by the nonlinear response of a thin glass substrate (1 mm).

### 4.5 Numerical Simulation

The LSPR excitation spectra of NT and the optical profiles of the OPDs with or without NT were analyzed by using COMSOL Multiphysics. The NT structure was meticulously modeled in COMSOL, including its core (PdCu), interlayer (Au), and outer shell (SiO_2_) components. The simulation setup positioned the NT within a large spherical medium (large enough to avoid physical reflections from the medium boundaries, while sufficiently small to enhance computational efficiency) possessing the dielectric constant of chloroform, representing the solvent environment during device fabrication. Through systematic parameter sweeps, the scattering characteristics of individual NTs were examined across a wavelength range of 300 to 1200 nm. This investigation involved varying the length (from 24 to 34 nm) and width (from 10 to 16 nm) in 1 nm increments. The scattering properties were calculated by determining the polarization-induced power dissipation under incident illumination, accounting for both longitudinal (in-plane) scattering components (*Ext*_l_) and transverse (out-of-plane) scattering components (*Ext*_t_). The LSPR peak position, corresponding to maximal electromagnetic field enhancement and energy dissipation, was identified mathematically when the first derivative of total extinction with respect to wavelength reached zero ( $\frac{d}{d\lambda }\left({Ext}_{l}+{Ext}_{t}\right)=0$ ) while the imaginary part of the metal’s permittivity (Im­(ε_metal_)) attained its extremum. The NT, with a width of 14 nm and a length of 30 nm, were optimized and examined for the performance of the OPDs with a layer configuration of ITO (150 nm)/PEDOT:PSS (40 nm)/BHJ (200 nm)/ PNDIT-F3N (10 nm)/Ag (120 nm). To enhance the reliability of the simulation, the mesh size of the BHJ area was set to 2 nm, and that of the other area was set to 20 nm, which is small enough to ensure accuracy and big enough to meet the calculating efficiency. All simulations were operated on a server (14th Gen Intel­(R) Core (TM) i9–14900K 3.20 GHz, 128 GB).

## Supplementary Material



## Notes

Extinction and absorption spectra; TEM and AFM images; size distributions; chemical structures; dark *J*–*V* characteristics and EQE spectra; *R* and *D** spectra; stability test; refractive indexes and extinction coefficients; light dependence analysis; trap density analysis; PL spectra; *C*–*V* characteristics; homemade image sensing system illustration; summary of performance of OPDs (PDF)
