# Supplementary material for: Enhanced Near-Infrared Organic Photodetectors Leveraging Core–Shell Nanotripods
Source: ACS Appl Mater Interfaces. 2025 Jun 2;17(23):34304–16. doi: 10.1021/acsami.5c02476 (PMC12163934; doi:10.1021/acsami.5c02476)
Supplement: Supplementary file 1 [file am5c02476_si_001.pdf]

Supporting Information

Enhanced Near-Infrared Organic Photodetectors Leveraging Core-Shell Nanotripods

Kaiwen Zheng[1], Baozhong Deng[1], Nan Chen[1], Clemence Chinaud-Chaix[2], Mona Tréguer-Delapierre[2], Bruno Grandidier[3], Renaud Bachelot[4], Tao Xu[1*], Jianhua Zhang[1], Furong Zhu[5*]

[1] School of Microelectronics, Shanghai University, 200444 Shanghai, China

[2] CNRS, Bordeaux INP, ICMCB, University of Bordeaux, UMR 5026, Pessac F-33600, France

[3] Université de Lille, CNRS, Centrale Lille, Université Polytechnique Hauts-de-France, Junia-ISEN, UMR 8520 - IEMN, 59000 Lille, France

[4] Light, nanomaterials, nanotechnologies (L2n) Laboratory, CNRS UMR 7076. University of Technology of Troyes, 12 rue Marie Curie, F-10004 Troyes Cedex, France, and CNRS-International-NTU-Thales Research Alliance (CINTRA), Nanyang Technological University. 50 Nanyang Drive, Singapore 637553, Singapore

[5] Department of Physics, Research Centre of Excellence for Organic Electronics, Institute of Advanced Materials, Hong Kong Baptist University, Kowloon Tong, Hong Kong, China

Emails: xtld@shu.edu.cn, frzhu@hkbu.edu.hk

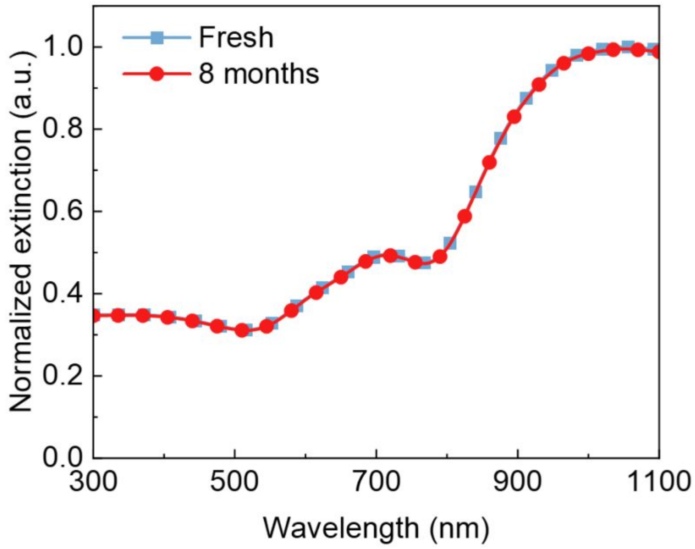

Figure S1. Extinction spectra of freshly prepared PdCu@Au@SiO_2 NT solution compared to the spectra after 8 months of storage in CF.

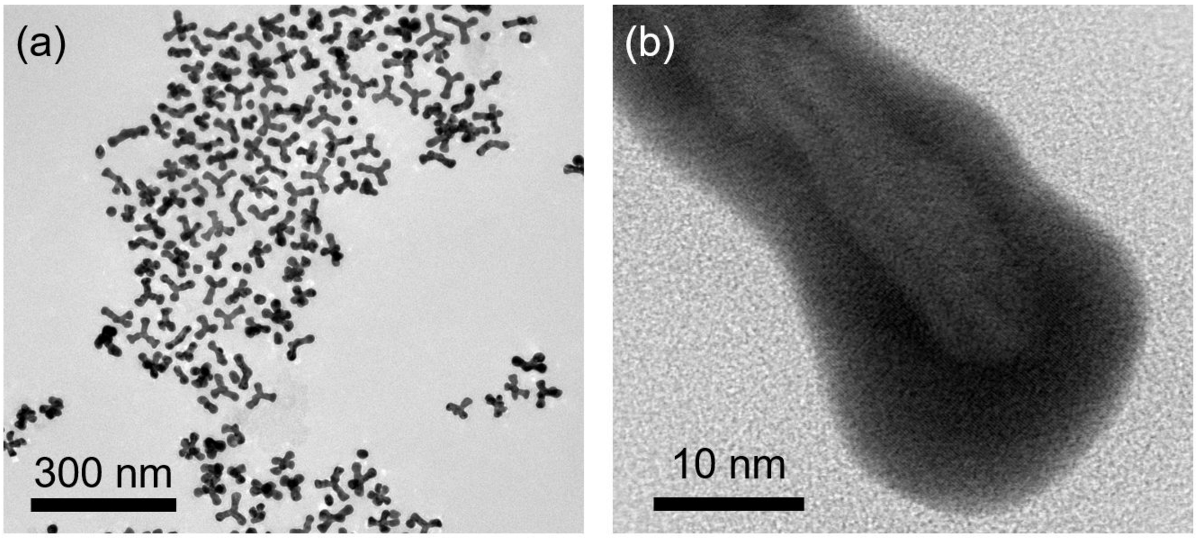

Figure S2. (a) TEM image of NT dispersed in CF. (b) HRTEM images of one branch of an NT.

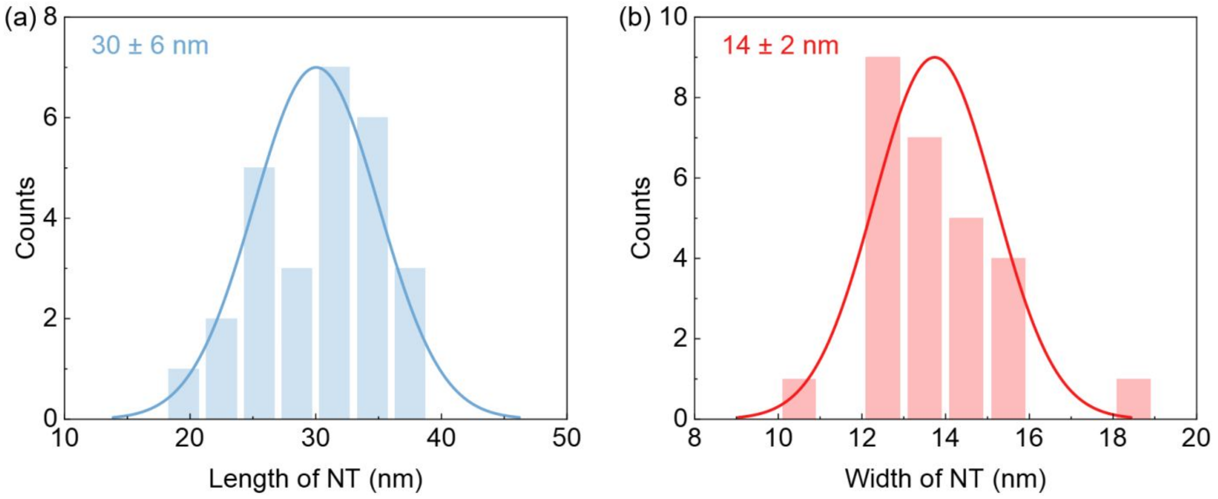

Figure S3. Size distribution of the NTs synthesized in this work: (a) branch length and (b) branch width of the core-shell PdCu@Au@SiO_2 NTs.

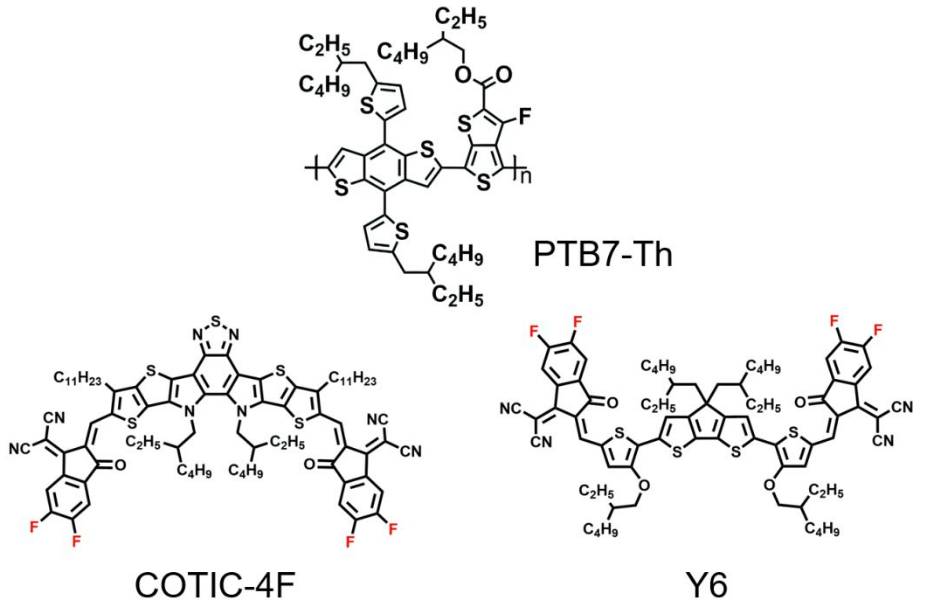

Figure S4. Molecular structures of the organic materials used in the ternary photoactive active layer.

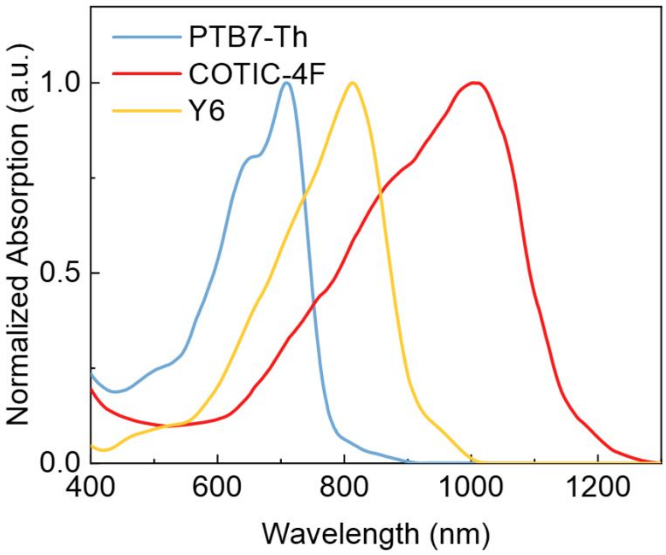

Figure S5. Normalized absorption spectra measured for the thin films of PTB7-Th, COTIC-4F, and Y6.

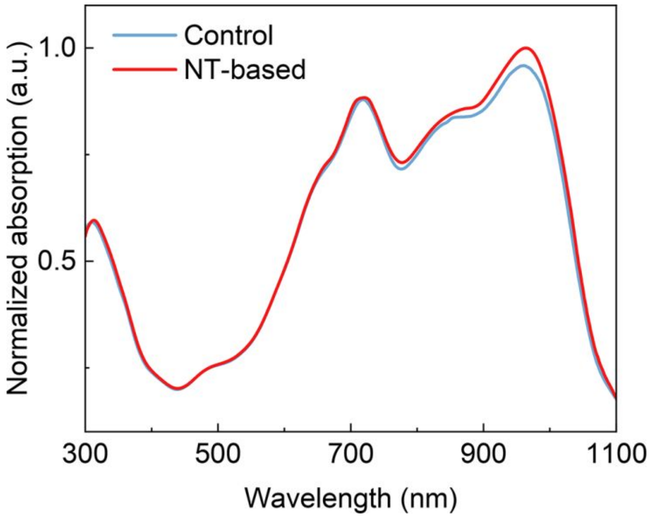

Figure S6. Normalized absorption spectra measured for the control BHJ film, prepared using the precursor solution with a weight ratio of PTB7-Th to COTIC-4F to Y6 of 1:1.125:0.375, and the NT-based BHJ film, prepared by incorporating a 0.15 wt% concentration of core-shell PdCu@Au@SiO_2 NTs into the precursor solution.

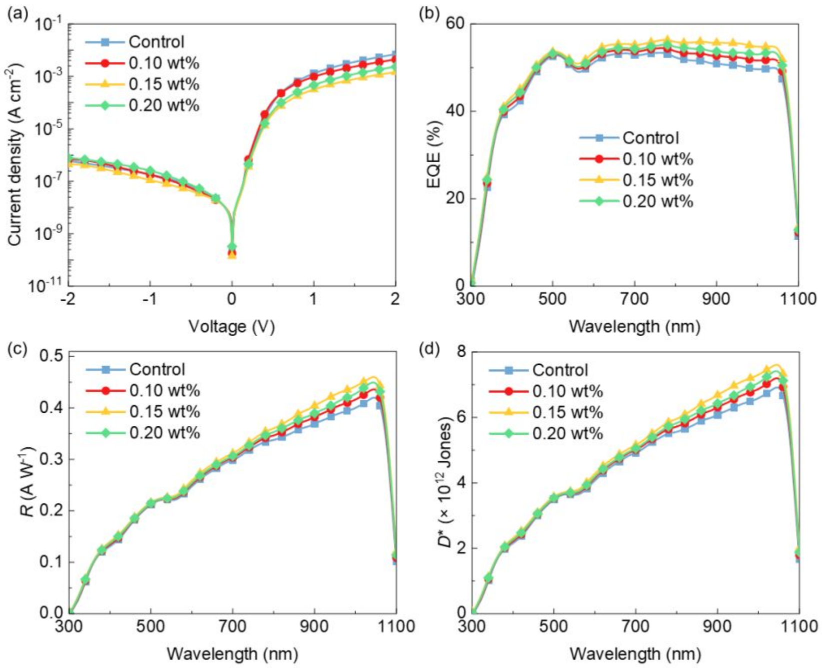

Figure S7. (a) J_d–V characteristics, (b) EQE spectra, (c) $R(\lambda)$, and (d) D^* spectra obtained for the control OPD and the NT-based OPDs incorporating different NT concentrations in the BHJ active layer, at -0.1 V.

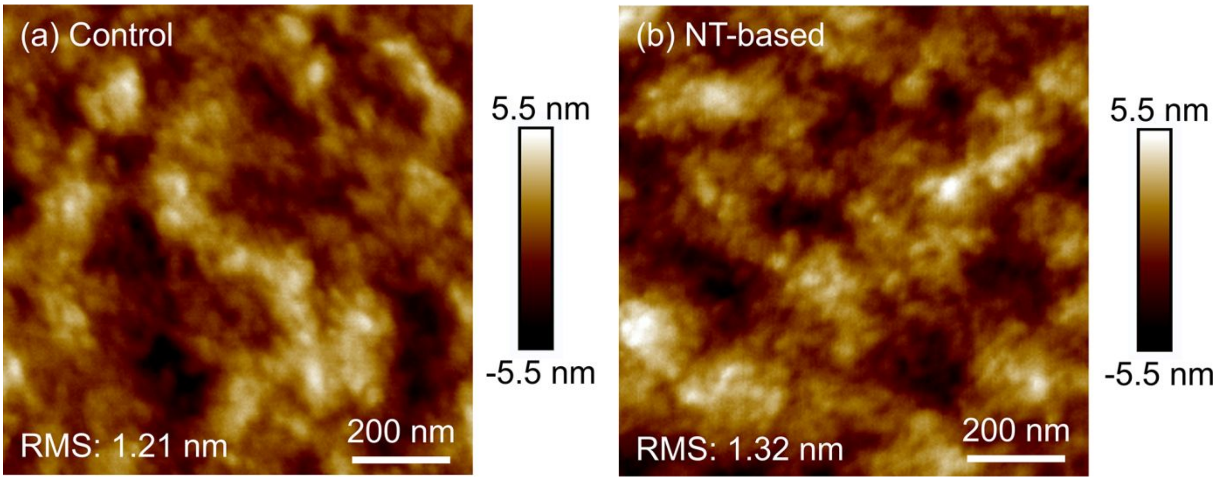

Figure S8. AFM images measured for (a) the control BHJ film, prepared using the precursor solution with a weight ratio of PTB7-Th to COTIC-4F to Y6 of 1:1.125:0.375, and (b) the NT-based BHJ film, prepared by incorporating a 0.15 wt% concentration of core-shell PdCu@Au@SiO_2 NTs into the precursor solution.

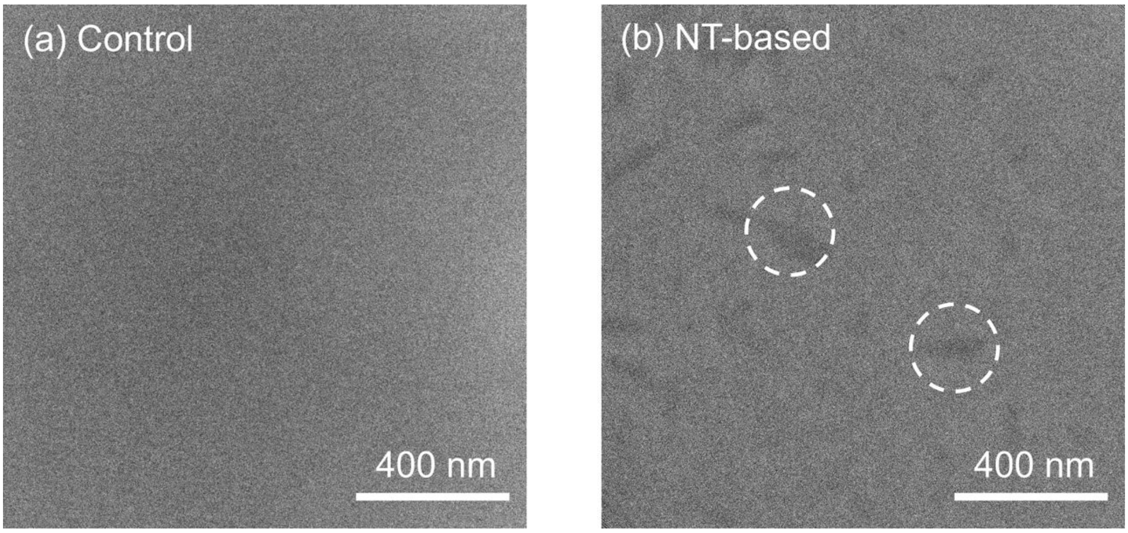

Figure S9. TEM images measured for (a) the control BHJ film, prepared using the precursor solution with a weight ratio of PTB7-Th to COTIC-4F to Y6 of 1:1.125:0.375, and (b) the NT-based BHJ film, prepared by incorporating a 0.15 wt% concentration of core-shell PdCu@Au@SiO_2 NTs into the precursor solution. The dashed circles indicate the locations of the NTs in the organic blend layer.

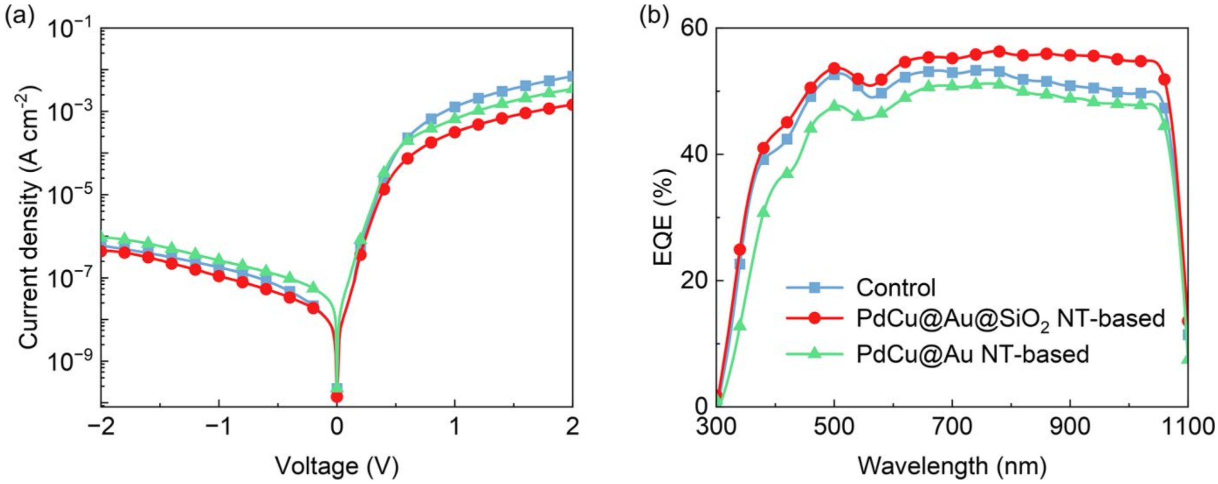

Figure S10. (a) J_d–V characteristics, and (b) EQE spectra measured for the NT-based and control OPDs incorporating a 0.15wt% concentration of core-shell PdCu@Au@SiO_2 NTs, operated at -0.1 V.

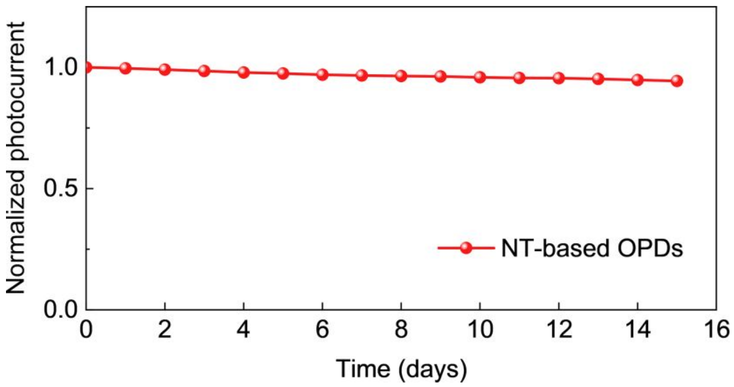

Figure S11. Aging test measured for the encapsulated NT-based OPDs over a period of 15 days in air.

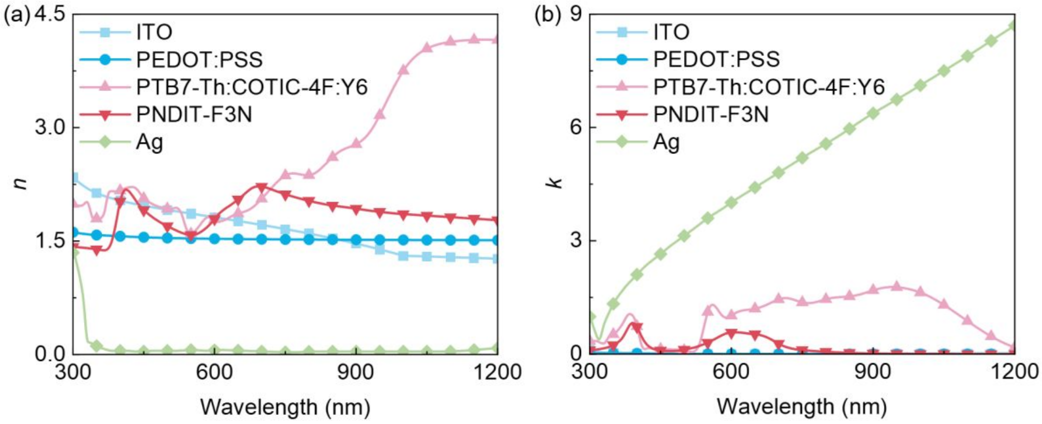

Figure S12. Optical constants: (a) refractive index and (b) extinction coefficient of all the functional materials used for the simulation.

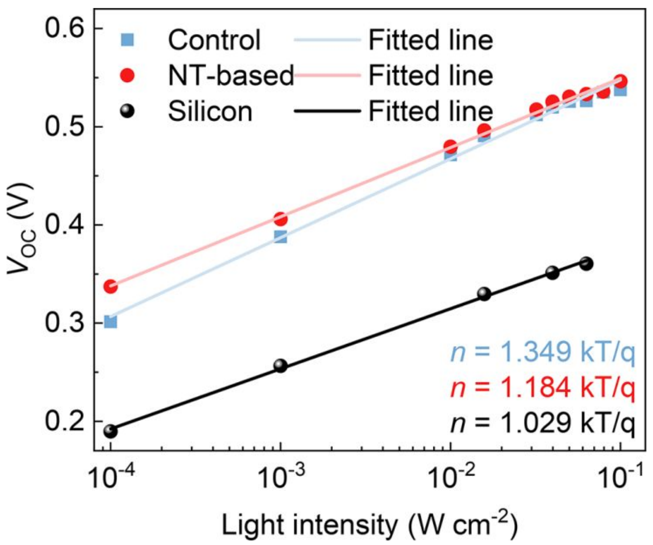

Figure S13. V_{OC}–P characteristics obtained for the control and NT-based OPDs, and a reference Si photodetector.

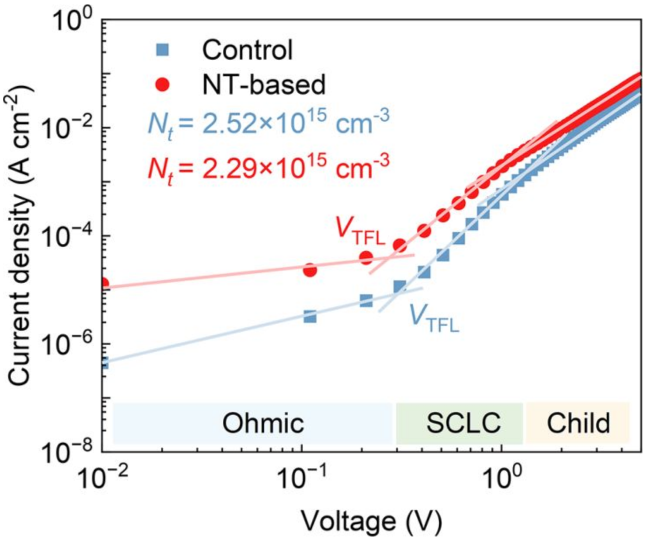

Figure S14. Double logarithmic J–V plots for the control and NT-based OPDs, illustrating the hole trap density, derived from the SCLC and Child regions. The solid lines represent the linear fitting for each OPD type, highlighting differences in trap density between the control and NT-based devices.

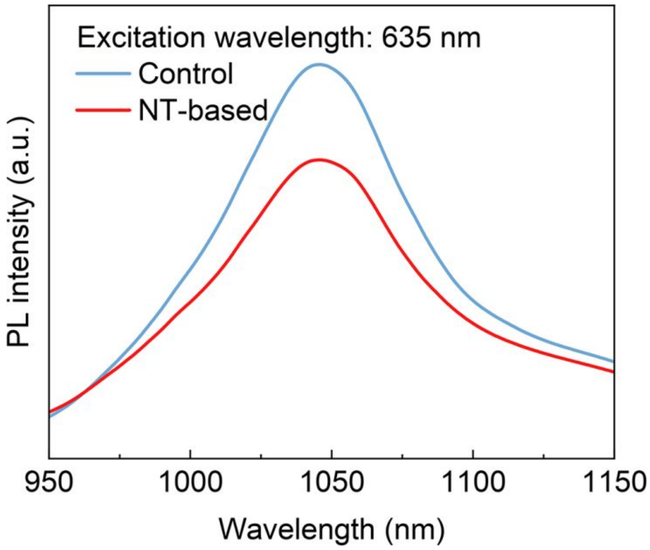

Figure S15. Normalized PL spectra measured for the control BHJ film, prepared using the precursor solution with a weight ratio of PTB7-Th to COTIC-4F to Y6 of 1:1.125:0.375, and the NT-based BHJ film, prepared by incorporating a 0.15 wt% concentration of core-shell PdCu@Au@SiO_2 NTs into the precursor solution.

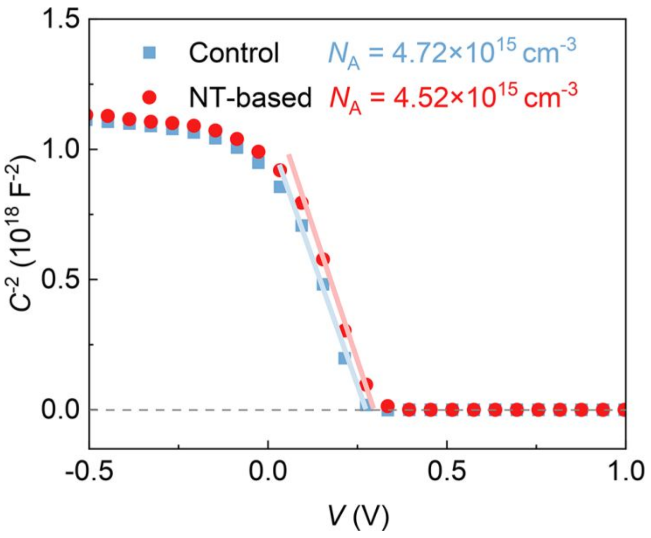

Figure S16. $1/C^2{-}V$ characteristics and Mott-Shockley plots for both the control and NT-based OPDs, with solid lines indicating the linear fitting for each device type.

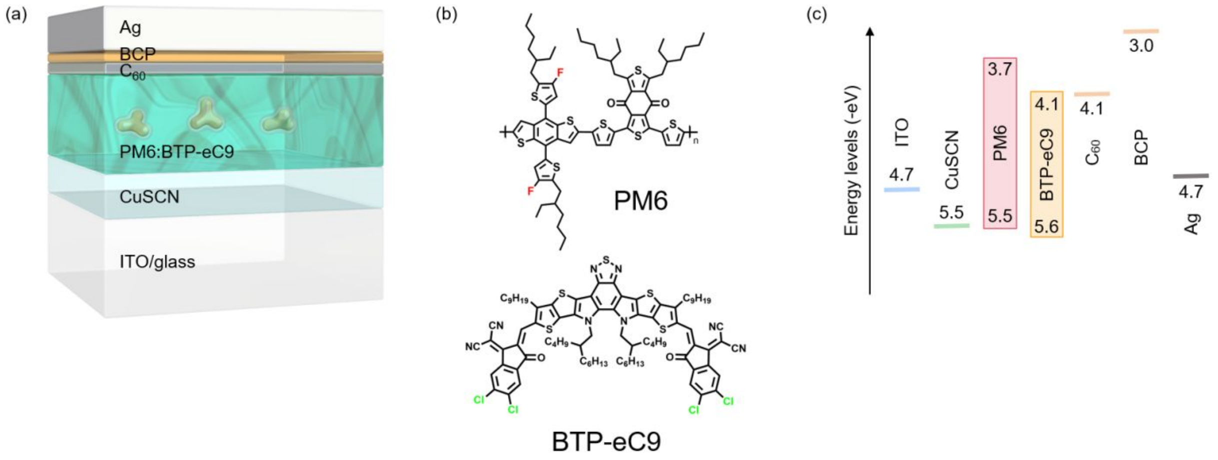

Figure S17. (a) Schematic cross-sectional view of a binary OPD. (b) Molecular structures of organic functional materials used in the binary active layer. (c) Schematic energy-level diagram of the functional materials used in the OPDs.

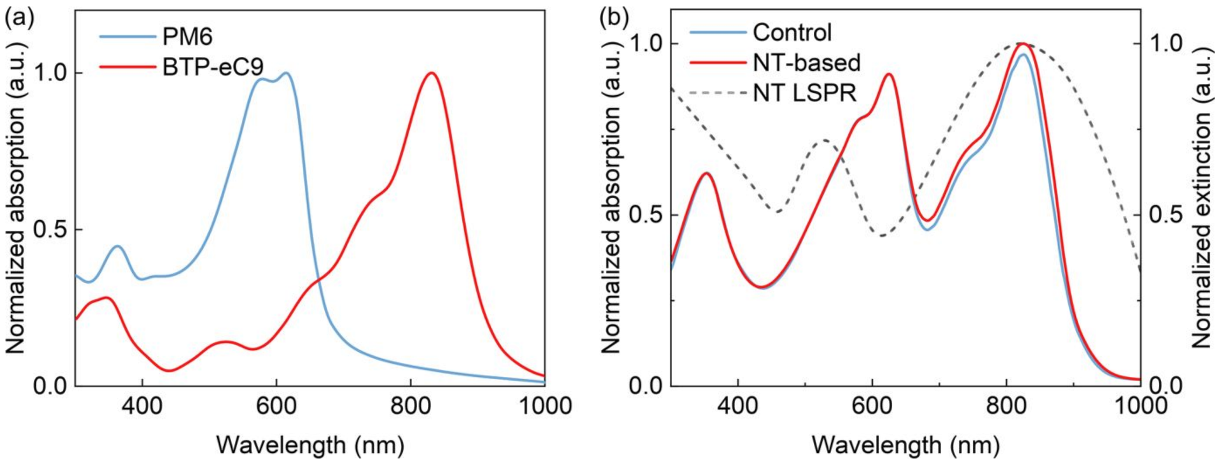

Figure S18. (a) Normalized absorption spectra measured for the thin films of PM6, and BTP-eC9. (b) Normalized absorption spectra measured for the control BHJ film, and the NT-based BHJ film, and normalized extinction spectra of NT in CF.

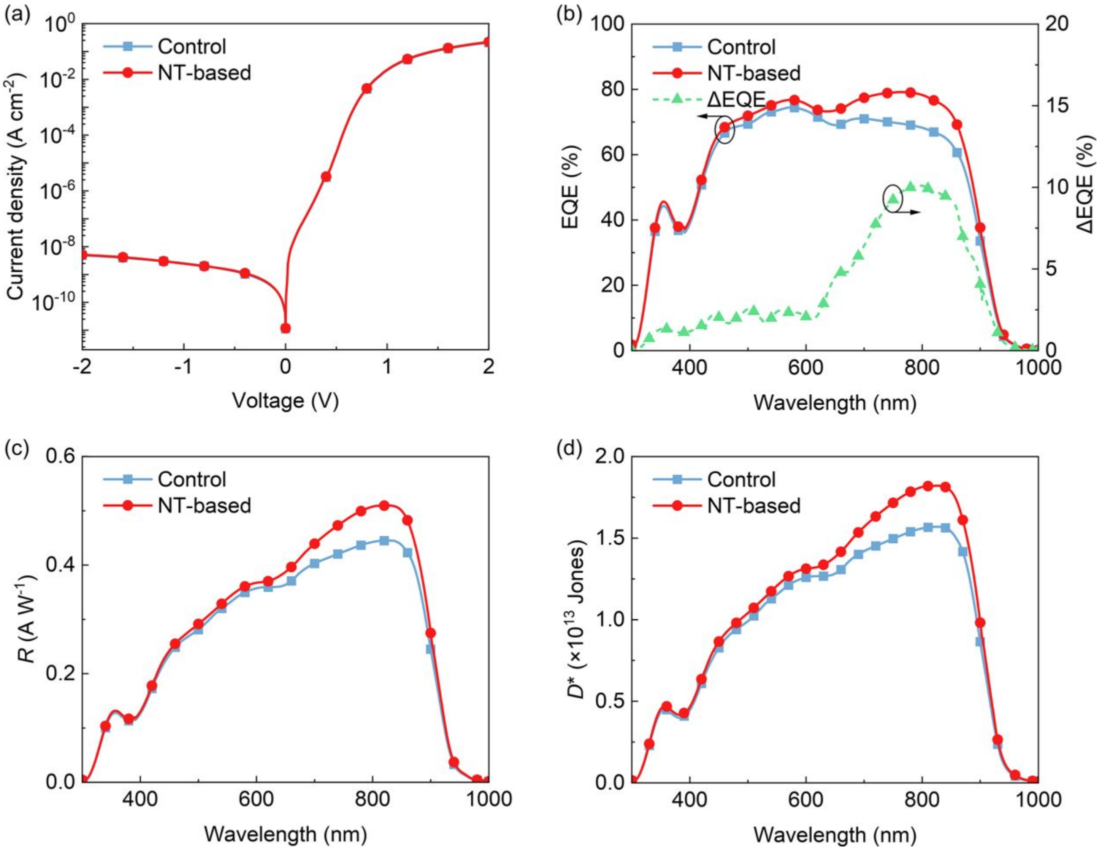

Figure S19. (a) J_d–V characteristics, (b) EQE spectra, (c) R spectra, and (d) D* spectra obtained for the NT-based and control binary OPDs operated without bias and operated at -1.0 V.

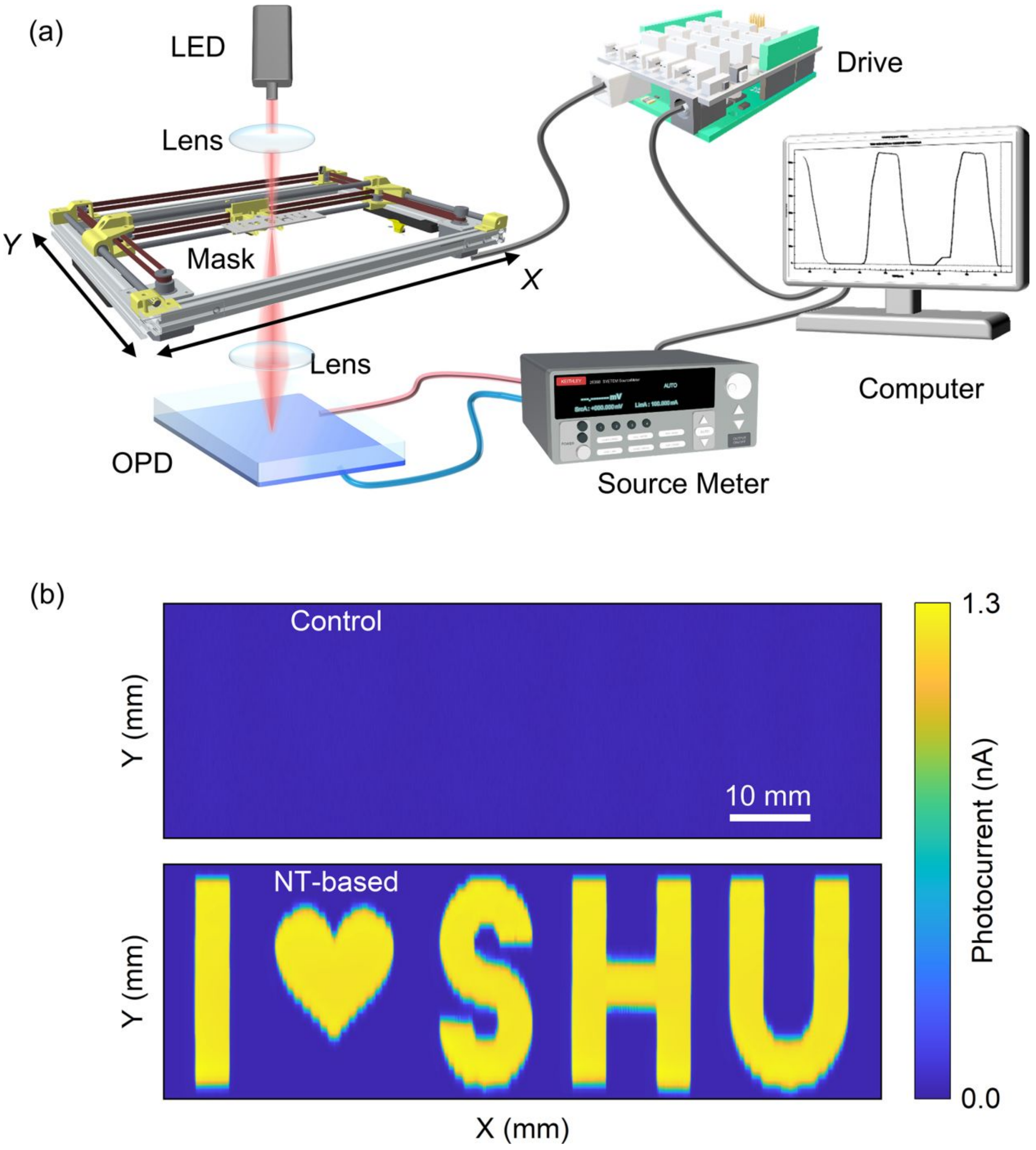

Figure S20. (a) Schematic diagram illustrating an image measurement system. (b) In this imaging setup, an LED light source is aligned with an NT-based OPD. The photocurrent is measured by moving the mask linearly along the X and Y directions, using a computer-controlled x-y stepper motor stage. This process generates the "SHU" images, which vary according to the light intensity.

Table S1. A summary of the LSPR peak position, optical effect, and the shape and size of the different metal nanostructures reported by different groups and in this work.

	LSPR peak position (nm)	Shape	Size (nm)	References
1	600	Sphere	59	*Adv. Optical Mater.* **2019**, *7*, 1801292
2	650-775	Sphere	120	*J. Phys. Chem. C* **2015**, *119*, 18635–18640
3	880 (with anisotropic scattering)	Rod	90	*Adv. Mater.* **2007**, *19*, 3771–3782
4	550	Disk	90	*Adv. Mater.* **2007**, *19*, 3771–3782
5	590	Triangle	15	*Adv. Mater.* **2007**, *19*, 3771–3782
6	650	Triangle	20	*Adv. Mater.* **2007**, *19*, 3771–3782
7	670 (with anisotropic scattering)	Rod	40	*ACS Nano* **2013**, *7*, 3815–3822
8	830 (with anisotropic scattering)	Rod	70	*ACS Nano* **2013**, *7*, 3815–3822
9	825 (with anisotropic scattering)	Bipyramid	100	*Adv. Sci.* **2022**, *9*, 2202150
	**1050 (with isotropic scattering)**	**Tripod**	**30**	**This work**
	**811 (with isotropic scattering)**	**Tripod**	**26**	**This work**

Table S2. Summary of the characterization results for the control OPD and the NT-based OPDs with different NT concentrations in the ternary BHJ active layer.

Device	EQE [%]	R [A/W]	J_d [A/cm^2]	D^* [Jones]
Control	49.47±0.32	0.42	(1.15±0.01) × 10^{-8}	6.90 × 10^{12}
0.10 wt%	51.34±0.19	0.43	(1.15±0.02) × 10^{-8}	7.18 × 10^{12}
0.15 wt% (optimal)	54.17±0.39	0.46	(1.14±0.01) × 10^{-8}	7.58 × 10^{12}
0.20 wt%	52.83±0.31	0.45	(1.15±0.02) × 10^{-8}	7.38 × 10^{12}

The results were obtained for the ternary OPDs operated at -0.1 V, under illumination of NIR (1050 nm) light, averaged from the measurement of 10 devices.

Table S3. The summary of charge carrier properties obtained for the control OPD and the NT-based OPDs with ternary BHJ active layer.

Device	P_{diss} [%]	P_{CC} [%]	μ_h [cm^2/V/S]	μ_e [cm^2/V/S]	μ_h/μ_e	V_{TFL} [V]	N_t [/cm^3]
Control	41.53	22.46	3.85×10^{-5}	2.28×10^{-5}	1.69	0.304	2.52×10^{15}
NT-based	47.19	25.61	4.30×10^{-5}	2.72×10^{-5}	1.58	0.276	2.29×10^{15}

Table S4. The summary of the device parameters obtained by the Mott-Shockley analysis.

Device	Slope [/F^2/V]	N_A [/cm^3]	V_{bi} [V]	W [nm]
Control	-3.99×10^{18}	4.72×10^{15}	0.274	139
NT-based	-4.16×10^{18}	4.52×10^{15}	0.295	147

Table S5. Summary of the characterization results for the control OPD and the NT-based OPDs with an optimal concentration of the NTs in the binary BHJ active layer.

Device	EQE [%]	R [A/W]	J_d [A/cm^2]	D^* [Jones]
Control	68.21±0.21	0.44	$(2.52\pm0.02) \times 10^{-9}$	1.56×10^{13}
NT-based	78.22±0.22	0.51	$(2.45\pm0.01) \times 10^{-9}$	1.81×10^{13}

The results were obtained for the binary OPDs operated at -0.1 V, under illumination of NIR (1050 nm) light, averaged from the measurement of 10 devices.
